# *Wolbachia* Symbiont Infections Induce Strong Cytoplasmic Incompatibility in the Tsetse Fly *Glossina morsitans*

**DOI:** 10.1371/journal.ppat.1002415

**Published:** 2011-12-08

**Authors:** Uzma Alam, Jan Medlock, Corey Brelsfoard, Roshan Pais, Claudia Lohs, Séverine Balmand, Jozef Carnogursky, Abdelaziz Heddi, Peter Takac, Alison Galvani, Serap Aksoy

**Affiliations:** 1 Yale University, School of Public Health, Division of Epidemiology of Microbial Diseases, New Haven, Connecticut, United States of America; 2 INSA-Lyon, UMR203 BF2I, INRA, Biologie Fonctionnelle Insectes et Interactions, Bat. Louis-Pasteur, Villeurbanne, France; 3 Institute of Zoology, Section of Molecular and Applied Zoology, Slovak Academy of Sciences, Bratislava, Slovakia; Stanford University, United States of America

## Abstract

Tsetse flies are vectors of the protozoan parasite African trypanosomes, which cause sleeping sickness disease in humans and nagana in livestock. Although there are no effective vaccines and efficacious drugs against this parasite, vector reduction methods have been successful in curbing the disease, especially for nagana. Potential vector control methods that do not involve use of chemicals is a genetic modification approach where flies engineered to be parasite resistant are allowed to replace their susceptible natural counterparts, and Sterile Insect technique (SIT) where males sterilized by chemical means are released to suppress female fecundity. The success of genetic modification approaches requires identification of strong drive systems to spread the desirable traits and the efficacy of SIT can be enhanced by identification of natural mating incompatibility. One such drive mechanism results from the cytoplasmic incompatibility (CI) phenomenon induced by the symbiont *Wolbachia*. CI can also be used to induce natural mating incompatibility between release males and natural populations. Although *Wolbachia* infections have been reported in tsetse, it has been a challenge to understand their functional biology as attempts to cure tsetse of *Wolbachia* infections by antibiotic treatment damages the obligate mutualistic symbiont (*Wigglesworthia*), without which the flies are sterile. Here, we developed aposymbiotic (symbiont-free) and fertile tsetse lines by dietary provisioning of tetracycline supplemented blood meals with yeast extract, which rescues *Wigglesworthia*-induced sterility. Our results reveal that *Wolbachia* infections confer strong CI during embryogenesis in *Wolbachia*-free (*Gmm^Apo^*) females when mated with *Wolbachia*-infected (*Gmm^Wt^*) males. These results are the first demonstration of the biological significance of *Wolbachia* infections in tsetse. Furthermore, when incorporated into a mathematical model, our results confirm that *Wolbachia* can be used successfully as a gene driver. This lays the foundation for new disease control methods including a population replacement approach with parasite resistant flies. Alternatively, the availability of males that are reproductively incompatible with natural populations can enhance the efficacy of the ongoing sterile insect technique (SIT) applications by eliminating the need for chemical irradiation.

## Author Summary

Infections with the parasitic bacterium *Wolbachia* are widespread in insects and cause a number of reproductive modifications, including cytoplasmic incompatibility (CI). There is growing interest in *Wolbachia,* as CI may be able to drive desired phenotypes such as disease resistance traits, into natural populations. Although *Wolbachia* infections had been reported in the medically and agriculturally important tsetse, their functional role was unknown. This is because attempts to cure tsetse of *Wolbachia* by antibiotic treatment damages the obligate mutualist *Wigglesworthia*, without which the flies are sterile. Here we have succeeded in the development of *Wolbachia* free and still fertile tsetse lines. Mating experiments for the first time provides evidence of strong CI in tsetse. We have incorporated our empirical data in a mathematical model and show that *Wolbachia* infections can be harnessed in tsetse to drive desirable phenotypes into natural populations in few generations. This finding provides additional support for the application of genetic approaches, which aim to spread parasite resistance traits in natural populations as a novel disease control method. Alternatively, releasing *Wolbachia* infected males can enhance Sterile Insect applications, as this will reduce the fecundity of natural females either uninfected or carrying a different strain of *Wolbachia*.

## Introduction

Tsetse flies are the sole vector of Human African Trypanosomiasis (HAT), also known as sleeping sickness, caused by the protozoan *Trypanosoma brucei spp.* in sub-Saharan Africa. Recent figures released by the World Health Organization (WHO) indicate that the devastating HAT epidemics, which started in the early 1990s, are coming under control and may no longer represent a major public health crisis [1]–[3]. While this news is welcoming, about 60 million people continue to live in tsetse infested areas at risk for HAT in 37 countries, and those at high risk are in remote areas where disease control is difficult to implement [2]. Diseases caused by trypanosomes in animals continue to be rampant in Africa and result in severe economic and nutritional losses. The ability to curb infections in animals stands to increase both economic and nutritional status of the continent.

Unfortunately, the disease toolbox remains very limited. To date, no vaccines have been developed for HAT, therapeutic treatments are expensive and have serious side effects, and diagnostic tools are inadequate [1]. Reduction of tsetse populations, however has proven as an effective method for disease control [1]. Although effective, implementation of vector control methods in remote regions of Africa where the disease is rampant is difficult, expensive and relies on extensive community participation and thus has not been widely exercised for human disease control [4]. During an endemic period however, vector control can be particularly advantageous in the absence of continued active case surveillance [5]. Mathematical models indicate that parasite infection prevalence in the tsetse host is an influential parameter for HAT epidemiology and disease dynamics [5]. Thus, reducing vector populations or reducing the parasite transmission ability of flies can be most effective in preventing disease emergence.

Advances in tsetse biology offer novel strategies, one being a population replacement approach to modify tsetse’s parasite transmission ability (vector competence) by expressing trypanocidal molecules in the gut bacterial symbiont fauna, termed paratransgenic transformation strategy [6]–[10]. For the paratransgenic approach to be successful, gene drive mechanisms need to be discovered to spread parasite resistant phenotypes into natural populations. An alternative vector control approach currently being entertained on the continent involves a population eradication method, through sterile male releases (SIT) [11]. Genetic methods that induce reproductive male sterility are superior to the currently available SIT strategy that relies on chemical irradiation to induce male sterility.

Tsetse flies are infected with multiple bacterial symbionts. Two of the symbionts are enteric: the obligate *Wigglesworthia glossinidia* reside within bacteriocytes in the midgut bacteriome organ as well as in milk gland accessory tissue [12], while commensal *Sodalis glossinidius* reside both inter- and extra-cellularly in various tissues [13]. A large portion of *Wigglesworthia’s* proteome encodes vitamin products that may be necessary to supplement the strictly vertebrate blood meal diet of tsetse [14]. Without the bacteriome population of *Wigglesworthia,* tsetse flies have reduced egg development and are infecund [15]–[18]. The third symbiont, *Wolbachia* resides mainly in the reproductive tissues [13], [19], [20].

Tsetse females have an unusual viviparous reproductive biology. Females develop a single oocyte per gonotrophic cycle. The oocyte is ovulated, fertilized and undergoes embryonic development in-utero. The resulting larva hatches and is carried in the intrauterine environment through three larval instars before being deposited. During its intrauterine life, the larva receives all of its nutrients in the form of milk secreted by the female accessory glands, milk glands. While *Wolbachia* is transovarially transmitted, the enteric symbionts are maternally transmitted into tsetse’s intrauterine larva through mother’s milk secretions [14]. By providing ampicillin in the blood meal diet, it has been possible to clear the extracellular *Wigglesworthia* in the milk without damaging the intracellular *Wigglesworthia* in the bacteriome [21]. Thus, such females remain fecund but give rise to sterile progeny that lack *Wigglesworthia* (both bacteriome and milk gland populations) but retain *Wolbachia* and *Sodalis*. As a result of the obligate role of *Wigglesworthia*, it has not been possible to use tetracycline treatment to cure *Wolbachia* infections, and the biological significance of *Wolbachia* infections in tsetse has thus remained elusive.

*Wolbachia* infections associated with various insects have been shown to cause a number of reproductive modifications in their hosts, the most common being CI [22]–[24]. CI occurs when a *Wolbachia* infected male mates with an uninfected female, causing developmental arrest of the embryo. In contrast, *Wolbachia* infected females can mate with either an uninfected male or a male infected with the same *Wolbachia* strain and produce viable *Wolbachia* infected offspring. This reproductive advantage of infected females results in the spread of *Wolbachia* infections along with other traits that infected insects may exhibit [25], [26]. Empirical studies and previously developed models have shown that the reproductive advantage provided by *Wolbachia* may be able to drive desired phenotypes along with other maternally inherited genes, organelles and/or symbionts into natural populations [27]–[30]. The *Wolbachia* type found in the tsetse species *Glossina morsitans morsitans* belongs to the *Wolbachia* A super group [20]. In a number of insect systems, *Wolbachia* strains belonging to the A super group have been associated with the CI phenotype in the different hosts they infect [31].

Here we investigated the possible role of *Wolbachia* symbionts that can be used to drive desirable tsetse phenotypes into natural populations, or to induce natural reproductive male sterility for field applications. We developed a dietary supplementation method that can restore fecundity of tsetse in the absence of their natural symbiotic fauna, including obligate *Wigglesworthia* and *Wolbachia*. We report on the fitness parameters of the engineered symbiont-free lines and on the level of CI expression after wild type and aposymbiotic flies are crossed. A mathematical model was also developed to ascertain whether *Wolbachia* infections in tsetse could be used to drive a disease refractory phenotype into a natural population.

## Results

### Dietary Supplementation with Yeast Extract Rescues Fecundity in the Absence of the Obligate *Wigglesworthia*

In many insect systems, tetracycline supplemented diet is used to generate *Wolbachia* free lines to demonstrate the functional role of *Wolbachia* through mating experiments. Inseminated tsetse females maintained on tetracycline-supplemented blood meals however do not generate any viable progeny. This is because tetracycline treatment damages the obligate intracellular *Wigglesworthia* present in the midgut bacteriome structure (Figure S1) [21]. These results are similar to prior reports where damage to *Wigglesworthia* had been found to reduce host fecundity [17], [21], [32].

**Figure 1:**
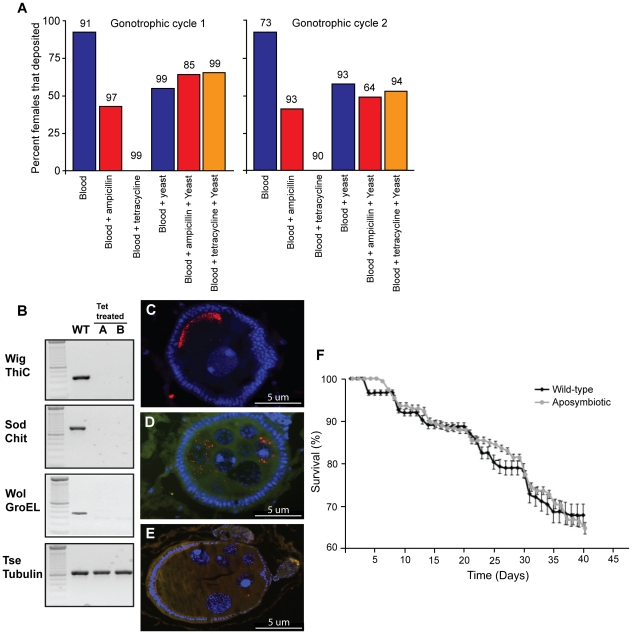
The effects of antibiotic treatment on *G. m. morsitans.* (A) Effect of yeast supplementation on percent larval deposition over two gonotrophic cycles between wild type flies maintained on normal blood supplemented with antibiotics (ampicillin or tetracycline) compared to flies maintained on yeast supplementation. The sample size (n) is above each column, and is represented as the number of females alive at the beginning of each gonotrophic cycle. (B) PCR analysis shows the *Gmm^Wt^* flies are positive for *Wigglesworthia* (Wig Thic), *Sodalis* (Sod Chit) and *Wolbachia* (Wol Groel). In contrast offspring resulting from tetracycline treated females (A and B) lack all three of the symbionts. The bottom panel shows gDNA quality as measured by tsetse β-tubulin. (C) Presence of *Wolbachia* infections in late developing egg chambers of *Gmm^Wt^* females. Nuclei are indicated by the blue DAPI stain and *Wolbachia* is shown by the red stain (D&E) Presence and absence of *Wolbachia* infections in early developing egg chambers of *Gmm^Wt^* and *Gmm^Apo^* females respectively. (F) Comparison of adult longevity between female *Gmm^Wt^* and *Gmm^Apo^* over a forty-day period on yeast supplemented diet. Error bars are reflective of standard error. Data points are offset for clarity.

The fecundity of fertile females maintained on various diets was evaluated (Figure 1A). Specifically, the diet combinations were as follows: (a) blood only, (b) blood and ampicillin, (c) blood and tetracycline, (d) blood and yeast, (e) blood, ampicillin and yeast, and (f) blood, tetracycline and yeast. We monitored the number of larva deposited in each group over a 40-day period when females undergo two gonotrophic cycles (defined as time required for the development of a single progeny in-utero). Under optimum conditions the first gonotrophic cycle takes about 20–22 days for development from egg to parturition. In subsequent gonotrophic cycles females produce a larva every 9 to 11 days. As we had previously shown, ampicillin treatment does not reduce fecundity since it does not damage *Wigglesworthia* resident within bacteriocytes in the midgut, unlike tetracycline, which clears all bacteria including *Wigglesworthia* and *Wolbachia* and induces sterility. Accordingly, ampicillin-receiving flies remained fecund while tetracycline receiving flies were rendered sterile.

Yeast extract (10% w/v) provisioning of the blood meal rescued fecundity of the females receiving tetracycline to similar levels as that of wild type and ampicillin receiving flies (65%, 55% and 64% over the first gonotrophic cycle and 53%, 58% and 49% over the second gonotrophic cycles, respectively). However, yeast provisioning at 10% w/v had a cost on fecundity when compared to flies maintained on normal blood meals, (92% versus 55% over the first gonotrophic cycle and 92% and 58% over the second gonotrophic cycle, respectively). Nevertheless, yeast supplementation was able to rescue the tetracycline-induced sterility to levels comparable to those observed for *Gmm^Wt^* receiving yeast or ampicillin supplemented blood meals, respectively (Figure 1A). Thus yeast supplemented dietary regiment allowed us to develop two lines to analyze the functional role of *Wolbachia* symbionts in tsetse biology; one lacking all symbionts (*Gmm^Apo^*) and another lacking *Wigglesworthia* but still retaining *Wolbachia* and *Sodalis* (*Gmm^Wig−^*).

The *Gmm^Apo^* progeny resulting from the first and second depositions of tetracycline treated mothers were tested for the presence of *Sodalis, Wigglesworthia* and *Wolbachia* by a bacterium-specific PCR-assay. The PCR-assay demonstrated the absence of all three symbionts as early as the first deposition in both the male and female *Gmm^Apo^* adults (Figure 1B). The absence of *Wolbachia* from the reproductive tissues of *Gmm^Apo^* females was also verified by Fluorescent In Situ Hybridization (FISH) analysis (Figure 1E). In contrast, *Wolbachia* was present in egg chambers during both early and late developmental stages in *Gmm^Wt^* females (Figure 1C & D). For analysis of longevity, survivorship curves were compared using the Kaplan-Meier and log rank tests. Longevity of F1 *Gmm^Apo^* females was compared to that of *Gmm^Wt^* adults maintained on the same yeast-supplemented blood meal over 40 days (two-gonotrophic cycles). No difference (X^2^ = 0.71, df = 1, P = 0.4) was observed in survivorship comparisons between the two groups (Figure 1F).

The second line (*Gmm^Wig−^*) generated from ampicillin treated females still retain their *Wolbachia* and *Sodalis* symbionts, while lacking both *Wigglesworthia* populations as evidenced by FISH and PCR amplification analysis [21]. When maintained on yeast-supplemented blood, this line (similar to *Gmm^Apo^*) also did not display any longevity differences from the *Gmm^Wt^* adults sustained on the same diet.

### No Paternal *Wolbachia* Effect Evidence in Aposymbiotic Flies

Tetracycline treatment has been shown to have a negative impact on the fertility of *Drosophila simulans* males [33]. To determine if the fertility of *Gmm^Apo^* males is negatively affected, we mated *Gmm^Wt^* females with either *Gmm^Wt^* or *Gmm^Apo^* males and maintained all flies on yeast-supplemented blood meals. Larval deposition and eclosion rates from both crosses were compared using arcsin(sqrrt(x)) transformed data to ensure normality. No significant difference was observed between the crosses for two gonotrophic cycles (P>0.05) (Table 1). The mean larval deposition rate for *Gmm^Wt^* females crossed with *Gmm^Wt^* males was 0.68 and 0.65 for the first and second gonotrophic cycles respectively, while the mean larval deposition rate for *Gmm^Wt^* females crossed with *Gmm^Apo^* males was 0.87 and 0.89 for the first and second gonotrophic cycles, respectively (Table 1). Similarly, no difference in eclosion rates was observed between the two groups (P>0.05) (Table 2). Of the pupae obtained in the first gonotrophic cycle from the *Gmm^Wt^* cross, 82% underwent eclosion compared to 83% for the cross between *Gmm^Wt^* females and *Gmm^Apo^* males. For the second gonotrophic cycle, we observed 89% average eclosion for pupae from *Gmm^Wt^* crosses and 93% for pupae from *Gmm^Wt^* females crossed with *Gmm^Apo^* males (Table 2). Taken together, these results demonstrate the preservation of reproductive fitness in *Gmm^Apo^* males and rule out possible paternal effects of *Wolbachia* in tsetse.

**Table 1:** CI expression shown by average larval deposition rates in crosses between *Gmm^Apo^* females mated with *Gmm^Wt^* males.

Cross type			Larval deposition rate 1^st^ gonotrophic cycle	Larval deposition rate 2^nd^ gonotrophic cycle
♀ *Gmm^Wt^*	x	♂ *Gmm^Wt^*	0.68±0.01^ab^; *n = 108*	0.65±0.07^ab^; *n = 89*
♀ *Gmm^Wt^*	x	♂ *Gmm^Apo^*	0.87±0.06^a^; *n = 59*	0.89±0.16^a^; *n = 48*
♀ *Gmm^Apo^*	x	♂ *Gmm^Apo^*	0.61±0.20^ab^; *n = 49*	0.53±0.18^b^; *n = 26*
♀ *Gmm^Apo^*	x	♂ *Gmm^Wt^*	0.10±0.02^c^; *n = 44*	0^c^; *n = 38*
♀ *Gmm^Wig−^*	x	♂ *Gmm^Wig−^*	0.68±0.14^b^; *n = 53*	0.59±0.07^ab^; *n = 50*

Larval deposition rates for each gonotrophic cycle and each cross type replicate were determined by dividing the number of larvae deposited per day by the number of remaining females in the cage on the day of larviposition, and summing the values for each gonotrophic cycle. Mean deposition rate values with different superscripted letters are statistically different from each other (P<0.05) using Tukey-Kramer post hoc multiple comparison tests within each gonotrophic cycle, ie., a, b, and c are significantly different from each other, c but not a and b are different from ab). *n* was calculated by combining the total number of females alive when the first larva were deposited for the three replicates of each cross type. *Gmm^Wt^*  =  Wild-type flies with all three symbionts, *Gmm^Apo^  = * flies treated with tetracycline that lack *Wigglesworthia*, *Sodalis*, and *Wolbachia*, and *Gmm^Wig−^*  =  flies treated with ampicillin that lack only *Wigglesworthia.*

**Table 2:** Eclosion rates (%) of deposited pupae.

Cross type			% Pupal Eclosion 1^st^ gonotrophic cycle	% Pupal Eclosion 2^nd^ gonotrophic cycle
♀ *Gmm^Wt^*	x	♂ *Gmm^Wt^*	82±8.0^a^; *n = 67*	89±5.0^a^; *n = 61*
♀ *Gmm^Wt^*	x	♂ *Gmm^Apo^*	83±9.0^a^; *n = 45*	93±6.0^a^; *n = 38*
♀ *Gmm^Apo^*	x	♂ *Gmm^Apo^*	60±18.0^ab^; *n = 34*	52±24.0^a^; *n = 27*
♀ *Gmm^Apo^*	x	♂ *Gmm^Wt^*	17±28.0^b^; *n = 4*	NA; *n = 0*
♀ *Gmm^Wig−^*	x	♂ *Gmm^Wig−^*	88±7.0^a^; *n = 33*	75±13^a^; *n = 25*

Mean % pupal eclosion values depicted by different superscripted letters are statistically different from each other (P<0.05) using Tukey-Kramer post hoc multiple comparison tests within each gonotrophic cycle, i.e., a and b are significantly different from each other, both not different from ab. *n*  =  the total number of pupae deposited.

### CI Expression

To determine the expression of *Wolbachia*-induced CI, cage population crosses were setup between *Gmm^Wt^* and *Gmm^Apo^* individuals. Cages were the experimental units and the data were arcsin(sqrrt(x)) transformed to ensure normality. To estimate the possible cost of reproductive fitness due to loss of *Wigglesworthia*, we made use of *Gmm^Wig−^* flies. Since *Gmm^Wig−^* flies still retained *Wolbachia* infections but lacked *Wigglesworthia* (as described earlier and in Figure 1A), this line served as the control for the CI cross in order to measure potential fecundity effects due to loss of *Wigglesworthia* in the *Gmm^Apo^* line and possible yeast-supplementation effects.

Although CI typically manifests itself as embryonic lethality, given the viviparous nature of reproduction in tsetse, we measured larval deposition rates, which are reflective of both successful embryogenesis and larvagenesis (Table 1). Differences in larval deposition rates (number of larva deposited per female) over the two gonotrophic cycles for all crosses were significant by ANOVA on arcsin(sqrrt(x)) transformed data (ANOVA; first deposition, F_4, 9_ = 20.6, P<0.0001, second deposition, F_4, 10_ = 21.9, P≤0.0001). No differences in larval deposition were observed between the crosses *Gmm^Wt^* × *Gmm^Wt^*, *Gmm^Wig^* ^−^ × *Gmm^Wig−^* and *Gmm* ^Apo^ × *Gmm* ^Apo^ (Table 1). However differences were observed in comparisons of the *Gmm^Apo^* × *Gmm^Wt^* cross with all other crosses for the first and second gonotrophic cycles (Table 1). Given that the *Gmm^Wig−^* females that lack *Wigglesworthia* are equally fecund as *Gmm^Wt^*, the strong incompatibility we observed in *Gmm^Apo^* females when crossed with *Gmm^Wt^* males is likely due to *Wolbachia* mediated reproductive affects, and not due to nutritional effects resulting from loss of the obligate symbiont *Wigglesworthia*.

We found that *Gmm^Wt^* females were compatible with all male infection types, while *Gmm^Apo^* females were only compatible with *Gmm^Apo^* males. Crosses of *Gmm^Apo^* females and *Gmm^Wt^* males demonstrated a pattern of unidirectional CI (Table 1). Spermathecae dissections of females in incompatible crosses that did not deposit a larva revealed the presence of sperm, suggesting females were inseminated and that lack of deposition was the result of CI. We also found that larval deposition rates and pupal eclosion rates showed similar patterns to large cage experiments when measured in single-pair crosses (Table S2). Differences were observed in larval deposition rates (number of larva deposited per female) over the two gonotrophic cycles for all single-pair crosses (Kruskal-Wallis; first deposition, χ^2^ = 9.3, df = 3, P = 0.03, second deposition, χ^2^ = 9.5, df = 3, P = 0.02). No differences in larval deposition were observed in pair-wise comparisons of the crosses *Gmm^Wt^* × *Gmm^Wt^*, *Gmm^Wt^* × *Gmm^Apo^* and *Gmm* ^Apo^ × *Gmm* ^Apo^ (Table S2). However differences were observed in comparisons of the incompatible *Gmm^Apo^* × *Gmm^Wt^* cross with all other crosses for the first and second gonotrophic cycles (Table S2). These results support strong CI expression driven by the *Wolbachia* infection status in female flies.

### Effect of Symbiont Infection on Host Eclosion

Other than reproductive modifications, *Wolbachia* infections have been shown to affect the fitness of their insect hosts [34], [35]. In this study, differences in eclosion rates (Table 2) were observed in the first gonotrophic cycle of crosses of *Gmm^Apo^*, *Gmm^Wt^, and Gmm^Wig−^* individuals on arcsin(sqrrt(x)) data (ANOVA, first gonotrophic cycle, F_4, 11_ = 7.5, P = 0.0036, second gonotrophic cycle, F_3, 8_ = 2.5, P = 0.13) (Table 2). No differences in eclosion rates were observed in single pair crosses for both gonotrophic cycles (Kruskal-Wallis; first gonotrophic cycle, χ^2^ = 0.74, df = 3, P = 0.86, second gonotrophic cycle, χ^2^ = 0.31, df = 2, P = 0.85) (Table S2). To determine if observed differences in eclosion rates were due to *Wolbachia* infection we compared the *Gmm^Wig−^* × *Gmm^Wig−^* and the *Gmm^Apo^* × *Gmm^Apo^* cross, since both strains lack *Wigglesworthia* infection, but one (*Gmm^Wig−^*) harbors *Wolbachia* infection. There were no significant differences however between these crosses (P>0.05) (Table 2), suggesting no extensive effect of *Wolbachia* infection on host eclosion rates.

### CI in Tsetse Manifests During Early Embryogenesis

**Figure 2:**
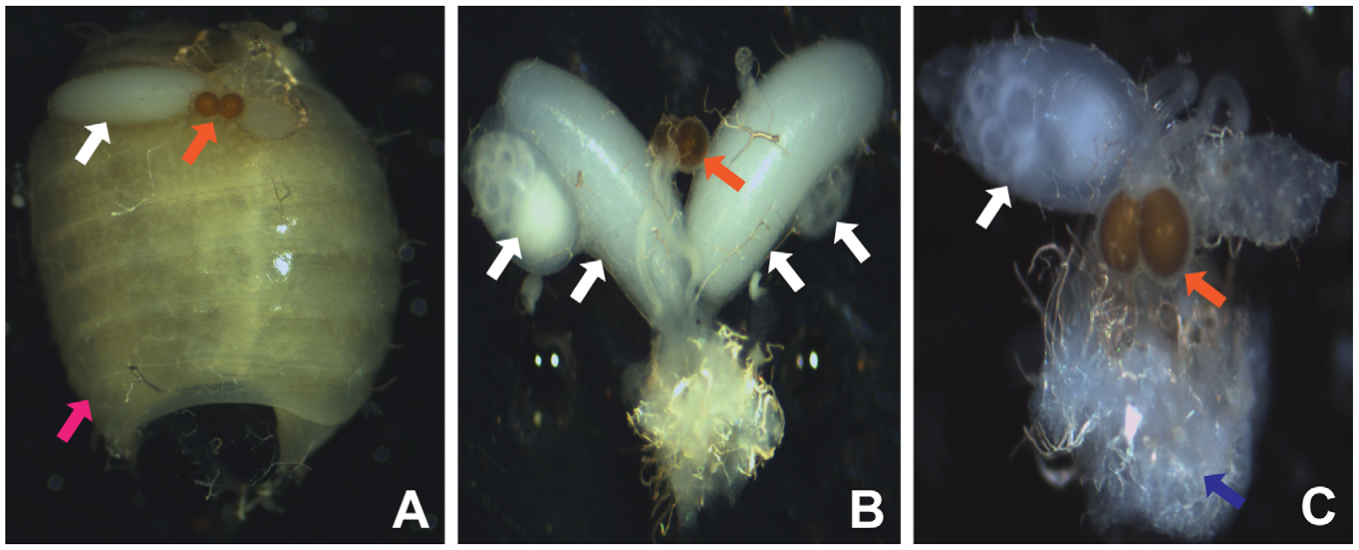
*Wolbachia*-induced CI phenotype in *G. m. morsitans*. Normal reproduction between *Gmm^Wt^* females and males is discernible by a developing oocyte indicated by the white arrow and the presence of a larva in the uterus indicated by the pink arrow, following the first gonotrophic cycle. (B) Unmated adult female tsetse. Unmated *Gmm^Wt^* females have an empty uterus and multiple developing oocytes indicated by white arrows. Note the transparent nature of the spermatheca reflective of lack of sperm (C) Manifested CI. CI is indicated by *Gmm^Apo^* females mated with *Gmm^Wt^* males by the absence of a larva in the uterus and deformed embryo indicated by the blue arrow. Many of these embryos were aborted without hatching into larva. Orange arrows indicate spermathecae in each image. Images were collected forty days (corresponding to the second gonotrophic cycle) post mating.

The CI phenotype was further examined by analyzing the reproductive tract physiology of tsetse females between incompatible and compatible crosses during embryonic development. Under normal conditions a single oocyte undergoes and completes oogenesis during larvagenesis. In compatible crosses (♀ *Gmm^Wt^* × ♂ *Gmm^Wt^*) we observed that the reproductive tract contains a developing larva in the uterus and a developing or completed oocyte in one of the two ovaries (Figure 2A). In an incompatible cross (♀ *Gmm^Apo^* × ♂ *Gmm^Wt^*) a developing oocyte is observed in one of the ovaries in the absence of a developing larva in the uterus, suggesting a disruption of embryogenesis or early larval development (Figure 2C). The observation of an incomplete oocyte in the absence of a developing larva in the uterus suggests the failure and abortion of either an embryo or very young larva. These observations differ from older *Gmm^Wt^* virgin females. Typically, *Gmm^Wt^* virgin females undergo oogenesis but do not undergo ovulation, which results in the development and eventual accumulation of two oocytes in each of the ovaries. Larvae are never observed in the uterus as developed oocytes are never ovulated, or fertilized in adult virgin females (Figure 2B).

### Spread of *Wolbachia* in Tsetse Populations

From the experimental data, we estimated the impact of CI on tsetse population biology using a Bayesian Markov chain Monte Carlo method. The transmission failure of *Wolbachia* from mothers to developing oocytes was moderate: 10.7% [0.07%, 22.7%] of progeny produced by *Gmm^Wt^* mothers were *Wolbachia* uninfected (Table 3). In addition, the incompatibility between *Gmm^Wt^* males and *Gmm^Apo^* females was strong: 79.8% [63.0%, 90.3%] of matings between *Gmm^Wt^* males and *Gmm^Apo^* females did not result in viable larvae as measured by pupal deposition. There was a significant fecundity (number of larval progeny deposited) benefit for *Wigglesworhia* infection: *Gmm^Wt^* females had 28.4% [8.5%, 54.2%] higher fecundity than *Gmm^Wig−^* females. Furthermore, *Wolbachia* infection alone was estimated to give a fecundity benefit of 19.3% [−9.2%, 57.9%]. This is an estimate of the fecundity difference between hypothetical females carrying *Wigglesworthia* and *Sodalis* but not *Wolbachia* and the experimental *Gmm^Wt^* females.

**Table 3:** Cytoplasmic-incompatibility parameter estimates.

Parameter	Median	95% Credible Interval
Fecundity Benefit of *Wolbachia* 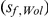	0.1925	[−0.0920, 0.5784]
Fecundity Benefit of *Wigglesworthia* 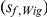	0.2839	[0.0854, 0.5420]
CI Strength 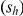	0.7976	[0.6295, 0.9025]
Transmission Failure 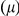	0.1073	[0.0069, 0.2274]

Shown are the posterior median and 95% credible interval from Bayesian Markov chain Monte Carlo estimation.

Most importantly, our model demonstrates that, given a large enough initial release, *Wolbachia* infected individuals will successfully invade a tsetse population (Table 4). The fixation prevalence of *Wolbachia* is estimated to be 96.9% [85.6%, 99.8%]. There may exist a release threshold, which an initial release must be above in order for *Wolbachia* to invade: the median was no release threshold (i.e. 0%), but the upper end of the 95% credible interval was a release of the size of 39.6% of the native population. The median threshold value is zero because, despite imperfect maternal transmission, the fecundity benefit of *Wolbachia* is strong enough to allow *Wolbachia* to invade a naïve tsetse population from any size initial release, no matter how small. In addition, the time to reach fixation from a release of the size of 10% of the native population can be relatively short: the median value was 529 days, however the upper end of the 95% credible interval was undefined because in more than 2.5% of samples, 10% initial release was below the release threshold.

**Table 4:** Population-genetics quantity estimates: the posterior median and 95% credible interval from Bayesian Markov chain Monte Carlo estimation.

Estimate	Median	95% Credible Interval
*Wolbachia* Fixation Prevalence	0.9689	[0.8559, 0.9984]
Release Threshold	0.0000	[0.0000, 0.3958]
Time to Fixation (days)	529	[296, ——]

*Wolbachia* fixation prevalence is the level at which *Wolbachia* is stably present in the population. Release threshold is the number of *Wolbachia*-positive tsetse that must be released into a *Wolbachia*-free population in order for *Wolbachia* to ultimately go to fixation, relative to the size of the existing population. Time to fixation is the number of days required to go from 10% initial *Wolbachia* prevalence to 95% of the fixation prevalence; its upper 95% CI is undefined because for more than 2.5% of samples, a release of 10% of the population is below the release threshold so that *Wolbachia* is driven from the population for these samples.

Sensitivity analysis showed that the model results are sensitive to both assumed and estimated parameters (supplementary material Text S1). In particular, time to fixation had the largest sensitivity to the time to first deposition and large elasticities to *Wolbachia*- and *Wigglesworthia*-related parameters, suggesting that improving the estimates of these parameters would most effectively improve the fidelity of the estimate of time to fixation.

## Discussion

Here, we report for the first time on the functional role of *Wolbachia* infections in tsetse, which support the expression of CI. Microscopic analyses of the CI expressing females show that loss of fecundity results from early embryogenic failure. Essential for our studies we have discovered that we can maintain *Wolbachia* cured tsetse lines fertile by dietary provisioning of tetracycline supplemented blood meals with yeast extract, despite the fact that such flies lack the obligate mutualist *Wigglesworthia*, which is essential for tsetse’s fecundity. When incorporated into a mathematical model, our results suggest that *Wolbachia* can be used successfully as a gene driver and, the time to reach fixation is relatively short given a large enough initial release: on the order of 1 to 2 years. These results provide a first insight into the role of *Wolbachia* infections in a viviparous insect and indicate that *Wolbachia* mediated CI can potentially be used to drive desirable tsetse phenotypes into natural populations.

Our data presented here as well as previous results from other studies indicate that in the absence of *Wigglesworthia*, tsetse females are rendered sterile. Our prior studies where we maintained inseminated flies on ampicillin supplemented blood diets resulted in progeny deposition. This is because ampicillin treatment did not affect the intracellular *Wigglesworthia* resident in the bacteriome organ in the midgut, which provides essential nutrients to maintain tsetse host fecundity [21]. Antibiotic ampicillin treatment however eliminated the extracellular *Wigglesworthia* population present in the milk gland essential for symbiont transmission, and thus the resulting progeny from such females lacked *Wigglesworthia (Gmm* ^Wig−^). Such progeny were reproductively sterile although they retained the symbiont *Wolbachia*. The tetracycline diet eliminated both intracellular and extracellular forms of *Wigglesworthia* and thus we did not obtain any viable progeny from inseminated females that were maintained on the tetracycline only diet. Prior studies showed that tetracycline blood meals supplemented with vitamin B1 could partially rescue fertility [15], but in our experiments vitamin supplementation could give rise to at most one progeny deposition, which either did not hatch or did not survive as an adult (data not shown). In sharp contrast, supplementation of the blood meal diet with 10%(w/v) yeast-extract reverted sterility in tetracycline treated flies to levels comparable to *Gmm^Wt^* and *Gmm* ^Wig−^ females receiving the same diet (Figure 1A). Although we have compared the fecundity of all three lines for two gonotrophic cycles here, yeast supplemented flies continue to deposit four to five progeny (data not shown). Given the complex nature of the yeast extract (peptides, amino acids, vitamins and other yeast cell components), it is difficult to know the exact nature of the essential nutrients it provides, but we believe that it could be working via supplementation of lipids and/or essential vitamins that are lacking in the strict blood diet of tsetse. However, we did observe some negative effect attributable to the yeast diet when the fecundity of *Gmm^Wt^* flies receiving yeast supplemented blood meals is compared to those receiving normal blood diets. As such, we are further investigating the use of different yeast supplementations and/or concentrations in an effort to improve the diet efficiency. Nevertheless the availability of *Wolbachia*-cured flies (*Gmm* ^Apo^) allowed us to begin to understand the functional role of this symbiosis.

In addition to *Wolbachia* symbiont specific PCR amplification, we confirmed the absence of *Wolbachia* from the reproductive tissues of *Gmm^Apo^* females by FISH analysis. We show the presence of *Wolbachia* in *Gmm^Wt^* females, isolates to a pole late in development (Figure 1C). There are a number of studies in other model systems that have investigated the link between *Wolbachia* localization during spermatogenesis and density effects on CI [36], [37]. However, other studies have found no correlation between *Wolbachia* density and CI during spermatogenesis [38], [39]. There have also been a number of studies investigating *Wolbachia* localization during oogenesis [40]–[42]. Different *Wolbachia* strains in *Drosophila* embryos display posterior, anterior, or cortical localization congruent with the classification based on the *wsp* gene sequence [39]. A positive correlation between levels of *Wolbachia* at the posterior pole and CI has been suggested, but this has yet to be examined in detail [42]. Not withstanding, assessing the role of *Wolbachia* during oogenesis is important, given that factors promoting CI rescue are deposited in the egg cytoplasm during oocyte development [43] and bacterial deposition in the oocyte is an essential even for efficient maternal transmission.

Before we could perform crossing experiments to assess for CI, we evaluated the effect of *Wolbachia* clearance on male reproductive capacity. This evaluation is important given that tetracycline has been shown to negatively affect reproductive fitness in *Drosophila simulans* [33]. Additionally, the importance of this finding is highlighted by a study of the mosquito *A. albopictus* system in which the natural *Wolbachia* strains (*w*AlbA and *w*AlbB) were cleared and transinfected with the *Wolbachia* strain wRi from *D. simulans* [44]. Their results showed that the *w*Ri transinfected males have a reduced mating capacity compared with the wild type super infected males [44]. In contrast, in our system, no decrease in mating capacity was observed in *Gmm^Apo^* males compared with *Gmm^Wt^* males under the laboratory conditions. Our observation agrees with the evolutionary model proposed by Charlat *et al*., [45], where *Wolbachia* is exclusively maternally transmitted therefore males may be considered an evolutionary dead end in terms of *Wolbachia* infection [46]. Consequently, no direct selection by *Wolbachia* can be theoretically expected on paternal reproductive fitness.

Loss of fecundity in the cross (♀ *Gmm^Apo^* x ♂ *Gmm^Wt^*) could conceivably arise from loss of *Wigglesworthia*-mediated nutritional benefits in *Gmm^Apo^* females rather than to *Wolbachia* mediated CI. To test this possibility, we compared the larval deposition rates in crosses between ♀*Gmm^Apo^* × ♂ *Gmm^Apo^* and ♀*Gmm^Wig−^* × ♂ *Gmm^Wig−^* flies (Table 1). Our results show no statistically significant differences between these crosses indicating that loss of fecundity in the CI cross is not due to loss of *Wigglesworthia*.

Our empirical results were used to parameterize a population genetic model of the spread of *Wolbachia*. Our model demonstrated that *Gmm^Wt^* would successfully invade an uninfected natural population with a large enough release given CI rates. Indeed, uninfected natural populations and natural populations with low infection prevalence have recently been identified for multiple tsetse species [47]. This modeling result is consistent with the natural spread of *Wolbachia* in *Drosophila* populations [48]–[50]. In addition, the rise to the predicted fixation prevalence of between 86% and 100% is rapid. Apparently, the *Wolbachia*-mediated CI has the potential to rapidly and effectively drive a desirable phenotype into natural populations. We have previously been able to culture and genetically transform the commensal symbiont of tsetse, *Sodalis glossinidius* [51]. It has also been possible to reintroduce the transformed *Sodalis* into tsetse, called a paratransgenic approach [52], [53]. Given that *Sodalis* resides in close proximity to pathogenic trypanosomes in tsetse’s midgut, products expressed in rec*Sodalis* can have an immediate effect on trypanosome biology. The potential paratransgenic strategy in tsetse could harness the *Wolbachia* mediated CI to drive a recombinant *Sodalis* strain that would encode parasite resistance genes into natural populations [6], [10]. Our studies on the maternal transmission dynamics of tsetse’s symbionts in the laboratory indicated perfect transmission of both *Wolbachia* and *Sodalis* into tsetse’s sequential progeny [54]. This high transmission fidelity of the two symbionts, coupled with strong nearly 100% CI caused by *Wolbachia* would serve paratransgenic applications favorably.

An alternative control strategy to paratransgenic population replacement strategy would be use CI as part of an incompatible insect technique (IIT), which is analogous to a SIT approach [29], [55]–[58]. In a *Wolbachia*-based SIT approach female sterility is artificially sustained by repeated releases of cytoplasmically incompatible males. Similar to SIT, the increasing ratio of incompatible matings over time can lead to population suppression. The benefit of an IIT strategy is that it would not require the use of irradiation or chemosterilants to sterilize males prior to release, which often reduces the fitness of released males, but would rely on the naturally induced sterility of an incompatible *Wolbachia* infection [59]. A *Wolbachia-*based paratransgenic and IIT control strategy for tsetse would rely upon the introduction of a novel infection type into a population with an existing infection that could result in bi-directional CI or the introduction of a novel infection into an uninfected host population. Typically, in other insect systems novel *Wolbachia* infections are established by embryonic microinjections [60], [61]. This would be difficult in tsetse given their viviparous reproductive biology, in that adult females carry and nourish their offspring for their entire larval developmental cycle making injections of embryos difficult. Future studies however can focus on the introduction of novel infection types via microinjection in aposymbiotic and naturally infected adult flies [62]. Maternal intrathoracic injections of *Wolbachia* infection establishment has also been successful in *Aedes aegypti* [63].

There has been a growing interest in understanding the variety of *Wolbachia* induced phenotypes in arthropods given the impact that *Wolbachia* infections could potentially have on genetic variation and host speciation impacting evolution of the species. Our data add to this growing field, as this is the first demonstration of the biological significance of *Wolbachia* infections in tsetse. Interestingly, CI in tsetse appears to be strong in that by the second gonotrophic cycle 0% of the females in an incompatible cross give rise to progeny. This is an exception given that in many insect systems incomplete CI is observed [27], [64]. Future studies with natural populations would now be important to confirm some of the parameters we report here including maternal transmission rates, infection prevalence and the maternal linkage efficacy between *Wolbachia* and other maternally transmitted symbionts such as *Sodalis,* which is being entertained for paratransgenic applications.

Additionally, the aposymbiotic lines generated in this study are currently being used to address the interactive role of trypanosome transmission in tsetse. The importance of which is highlighted by recent studies that have shown that *Wolbachia* infections may impact host immune biology, limiting pathogen proliferation in insect hosts [65]–[70].

## Materials and Methods

### Fly Rearing

The *Glossina morsitans morsitans* colony maintained in the insectary at Yale University was originally established from puparia collected in Zimbabwe. Newly emerged flies are separated based on sex and mated at three to four days post eclosion. Flies are maintained at 24±1°C with 50 – 55% relative humidity and fed defibrinated bovine blood (HemoStat Laboratories, CA) every forty eight hours using an artificial membrane system [71]. Selective elimination of natural tsetse endosymbionts was obtained as described below.

### Tetracycline Treatment

Wild type (*Gmm^Wt^*) fertile females were maintained on blood meals supplemented with 10% (w/v) yeast extract (Becton Dickinson) and 20 ug/ml of tetracycline. The yeast extract was briefly boiled in water before being added the blood meal each time. Flies were fed every 48 h using an artificial membrane feeding system (as above) for the duration of their life span. The resulting progeny are aposymbiotic (*Gmm^Apo^)* in that they lack their natural endosymbionts, *Wigglesworthia* and *Wolbachia.* These *Gmm^Apo^* lines were maintained on blood meals supplemented with 10% (w/v) yeast extract without tetracycline.

### Ampicillin Treatment

*Gmm^Wt^* fertile females were maintained on blood meals supplemented with 50 ug/ml of ampicillin. The resulting progeny do not have *Wigglesworthia (Gmm^Wig−^),* and were maintained on blood meals supplemented with 10% (w/v) yeast extract without ampicillin.

### Monitoring the Fecundity Cost of Yeast-extract Supplementation

Newly eclosed aged matched females and males were divided into six groups and copulation observed. Three of these groups were provided with either normal blood meals (control) or blood meals supplemented with ampicillin at 50 ug/ml or tetracycline at 20 ug/ml. Whereas the remaining three groups received blood meals supplemented with 10% (w/v) yeast extract with either ampicillin (50 ug/ml) or tetracycline (20 ug/ml). The cages were monitored daily for pupal deposition and fly mortality over two gonotrophic cycles (40 days). Fecundity was quantified by determining the number of fecund females relative to total number of females alive at the end of the gonotrophic cycle to give an average percent of females depositing pupae. Each group was setup with 100 females per cage.

### Symbiont Prevalence Assay

Total DNA was extracted from adults eight days post eclosion using the Qiagen Blood and Tissue extraction kit under manufacturers conditions (Qiagen Kit #, 69506. CA). The presence of the symbionts *Sodalis, Wigglesworthia* and *Wolbachia* was determined by a species-specific PCR amplification assay using the primer sets and conditions described (Table S1). For input DNA quality control, the tsetse gene β-*tubulin* (*GmmTub*) specific primer set was used. All PCR reactions were performed in an MJ-Research thermocycler and the amplification products were analyzed by electrophoresis on a 1% agarose gel and visualized using image analysis software.

### *Wolbachia* Infection Status by FISH

Dissected reproductive tracts from *Gmm^Wt^* and *Gmm^Apo^* females were fixed in 4% paraformaldehyde (PFA), embedded in paraffin, cut into 5 mm thick sections and mounted on poly _L_-lysine coated microscopy slides. After dewaxing in methylcyclohexane and rehydration the sections were processed using the FISH protocol previously described in Anselme *et al.* 2006 [72]. Slides were covered with a drop of 70% acetic acid and incubated at 45°C until drop had dried, followed by dehydration and a 10 min deproteinization step in 0.01N HCl/pepsine at 37°C. Slides were then dehydrated again, prehybridized for 30 min at 45°C and hybridized for 3 h at 45°C with 5′ end rhodamine labeled 16S RNA probes (5′-AAT CCG GCC GAR CCG ACC C -3′) and (5′-CTT CTG TGA GTA CCG TCA TTA TC -3′). Microscopic analyses were conducted using a Zeiss Axioskop2 microscope equipped with an Infinity1 USB 2.0 camera and software (Lumenera Corporation). Fluorescent images were taken using a fluorescent filter set with fluorescein, rhodamine and DAPI specific channels.

### Monitoring Longevity of *Gmm^Apo^* and *Gmm^Wt^* Females

*Gmm^Apo^* and *Gmm^Wt^* flies that emerged within a 24-hour period (teneral) were collected, mated with *Gmm^Apo^* males at a ratio of 5∶2 and copulation was observed. After six days males were removed from experimental cages. Six independent cages were set-up for both *Gmm^Apo^* and *Gmm^Wt^* groups, comprising of a total of 169 *Gmm^Apo^* and 170 *Gmm^Wt^* females, respectively. Both the males and females used represented offspring acquired from different gonotrophic cycles (1^st^ and 2^nd^). All flies were maintained on yeast extract supplemented blood meals and fly mortality was monitored daily over a 40-day period.

### CI Mating Crosses

To determine the expression of CI, reciprocal crosses were set up between *Gmm^Apo^*, *Gmm^Wt^* and *Gmm^Wig−^* flies, in triplicate. Cages with a minimum of 15 females and 7 males each were set-up in the following combinations: 1) ♀ *Gmm^Wt^* × ♂ *Gmm^Wt^, *2) ♀ Gmm^Wt^ × ♂ Gmm^Apo^, 3) ♀ Gmm^Apo^ × ♂ Gmm^Apo^, 4) ♀ Gmm^Apo^ × ♂ Gmm^Wt^ and 5) ♀ *Gmm^Wig−^* × ♂ *Gmm^Wig−^.* All flies received yeast supplemented blood meal diets. Flies were observed over two-gonotrophic cycles with daily recording of mortality, larval deposition dates, pupal eclosion dates and sex of emergent progeny. Larval deposition rates for each gonotrophic cycle were determined by dividing the number of larvae deposited per day by the number of remaining females in the cage on the day of larviposition and summing the values for each gonotrophic cycle. At the conclusion of the experiment, all females were checked for insemination by examination of dissected spermatheca for the presence of sperm microscopically. Additionally, single line crosses consisting of a single female and male per cage were set up (Table S2). For the ♀ *Gmm^Wt^* × ♂ *Gmm^Wt^* a total of 31 crosses were set up. Also set up were 40 crosses for ♀ *Gmm^Wt^* × ♂ *Gmm^Apo^*, 20 for ♀ *Gmm^Apo^* × ♂ *Gmm^Apo^* and 33 for ♀ *Gmm^Apo^* × ♂ *Gmm^Wt^*. Both the males and females used in these crosses represented offspring acquired from different gonotrophic cycles to rule out batch affects. Spermathecae of females was also dissected to confirm insemination.

### Mathematical Modeling

Here we will briefly describe the mathematical modeling used in this study; full details are available in the supplementary material (Text S1). The data from mating crosses were modeled as samples from the standard binomial random variable, with probability of larval deposition per mated female per gonotrophic cycle, and using a different probability for each cross. Following the empirical findings regarding *Wolbachia* -mediated CI in *Drosophila* [48], the probabilities were then defined in terms of four mechanistic parameters: the probability of reproduction success (larval deposit) from a cross between an *Gmm^Apo^* female and an *Gmm^Apo^* male (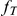), the proportion of *Wolbachia*-free eggs of *Wolbachia*-carrying mothers (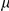), the relative benefit to reproduction success of *Wolbachia* infection to females (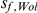), the relative benefit to reproduction success of *Wigglesworthia* infection to females (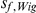), and the proportion of fertilizations of *Wolbachia*-free eggs by *Wolbachia*-affected sperm that are not viable (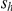). The larval-deposition probabilities in terms of these parameters are
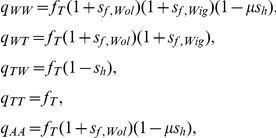
where the subscripts refer to the types of the female and male, respectively, with 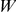 for wild type (*Gmm^Wt^*), 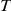 for tetracycline treated (*Gmm^Apo^*), and 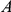 for ampicillin treated (*Gmm^Wig−^*).

In addition to these mechanistic parameters, we also estimated population-genetic quantities fundamental to the invasion of *Wolbachia* into a novel tsetse population. Again following existing models for *Wolbachia-*induced CI in *Drosophila* [38], a mathematical model was developed for the temporal evolution of tsetse abundance with and without *Wolbachia* infection. We incorporated the *Wolbachia*-mediated CI trade-off of the fitness cost to male hosts in reducing their mating success with uninfected females versus the fitness benefit to female hosts in allowing them to successfully mate with both infected and uninfected males (in addition to direct effects of *Wolbachia* on fecundity and mortality).

For some values of the mechanistic parameters, these models exhibit a threshold for *Wolbachia* invasion into the host population: if, in a novel population, the proportion that is initially *Wolbachia* infected is above the threshold, *Wolbachia* will continue to stable fixation in the population at a high level. If the proportion infected is below the threshold, *Wolbachia* will be driven out of the population over time. This threshold level was calculated, along with the prevalence of *Wolbachia* at fixation, and the time to fixation. For the population-genetic model, several parameters could not be estimated from the data on mating crosses. Thus, we also performed a sensitivity analysis on these parameters, along with the parameters estimated from the mating-cross data.

To estimate both the mechanistic parameters for CI and the population-genetics quantities derived from these parameters, a Bayesian Markov chain Monte Carlo (MCMC) method was used with uninformative prior distributions for the parameters [49].

## Supporting Information

Figure S1 The effect of antibiotics on the bacteriome of *G. m. morsitans*. Images of bacteriome sections stained with Giemsa (A) bacteriome organ showing bacteriocytes harboring *Wigglesworthia* from a female maintained on normal bloodmeals, image taken at 10x magnification (B) bacterioctes taken 40 magnification from a female maintained on ampicillin supplemented diet. A normal bacteriome structure is retained on the ampicillin diet allowing for continued fertility of such females. (C and D) Bacteriome structure observed in the progeny of ampicillin receiving females (C) and tetracycline and yeast extract receiving females in (D). In these individuals, the bacteriocyctes lack *Wigglesworthia* and these females are reproductively sterile, images taken at 10x magnification.
(TIF)

Table S1 Symbiont PCR primers.
(PDF)

Table S2 Larval deposition and pupal eclosion data for single cage crosses. In three separate experiments % larval deposition and % eclosion of the pupa deposited was determined. For each experiment, number of larval deposited for surviving females over two gonotrophic cycles and number of their pupae that hatched were recorded. Larval deposition was used as a measure of CI expression. To analyze for CI in replicate experiments of individual crosses, multiple Wilcoxon tests, with a Bonferroni correction were conducted to compare larval deposition rates. Wilcoxon tests, with a Bonferrroni correction were also conducted to compare pupal eclosion. Superscripted letters indicate significant differences, P<0.01.
(PDF)

Text S1 Mathematical methods.
(PDF)

## References

[1] Simarro PP, Jannin J, Cattand P (2008). Eliminating human African trypanosomiasis: where do we stand and what comes next?. PLoS Med.

[2] Cecchi G, Paone M, Franco J, Fevre E, Diarra A (2009). Towards the atlas of human African trypanosomiasis.. Int J Health Geogr.

[3] Simarro P, Diarra A, Ruiz Postigo J, Franco J, Jannin J (2011). The human African Trypanosomiasis control and surveillance programme of the World Health Organization 2000-2009.. PLoS Negl Trop Dis.

[4] Leak SG, Peregrine AS, Mulatu W, Rowlands GJ, D'Ieteren G (1996). Use of insecticide-impregnated targets for the control of tsetse flies (*Glossina spp*.) and trypanosomiasis occurring in cattle in an area of south-west Ethiopia with a high prevalence of drug-resistant trypanosomes.. Trop Med Int Health.

[5] Davis S, Aksoy S, Galvani A (2010). A global sensitivity analysis for African sleeping sickness..

[6] Aksoy S, Weiss B, Attardo G (2008). Paratransgenesis applied for control of tsetse transmitted sleeping sickness.. Adv Exp Med Biol.

[7] Chen XA, Aksoy S (1999). Tissue tropism, transmission and expression of foreign genes *in vivo* in midgut symbionts of tsetse flies.. Insect Mol Biol.

[8] Weiss BL, Mouchotte R, Rio RV, Wu YN, Wu Z (2006). Interspecific transfer of bacterial endosymbionts between tsetse fly species: infection establishment and effect on host fitness.. Appl Environ Microbiol.

[9] Welburn SC, Maudlin I, Ellis DS (1987). In vitro cultivation of *rickettsia*-like-organisms from *Glossina* spp.. Ann Trop Med Parasitol.

[10] Rio RV, Hu Y, Aksoy S (2004). Strategies of the home-team: symbioses exploited for vector-borne disease control.. Trends Microbiol.

[11] Vreysen MJ, Saleh KM, Ali MY, Abdulla AM, Zhu Z (2000). *Glossina austeni* (Diptera: Glossinidae) eradicated on the Island of Unguga, Zanzibar, using the sterile insect technique.. J Econ Entomol.

[12] Aksoy S (1995). *Wigglesworthia* gen. nov. and *Wigglesworthia glossinidia* sp. nov., taxa consisting of the mycetocyte-associated, primary endosymbionts of tsetse flies.. Int J Syst Bacteriol.

[13] Aksoy S (2000). Tsetse - A haven for microorganisms.. Parasitol Today.

[14] Attardo GM, Lohs C, Heddi A, Alam UH, Yildirim S (2008). Analysis of milk gland structure and function in *Glossina morsitans*: Milk protein production, symbiont populations and fecundity.. J Insect Physiol.

[15] Nogge G (1976). Sterility in tsetse flies (*Glossinia morsitans Westwood*) caused by loss of symbionts.. Experientia.

[16] Nogge G (1978). Apos-Symbiotic tsetse flies, *Glossina-Morsitans-Morsitans* obtained by feeding on rabbits immunized specifically with symbionts.. J Insect Physiol.

[17] Nogge G, Schwemmler W, Schenk H (1980). Elimination of symbionts of tsetse flies (*Glossina m. morsitans* Westw.) by help of specific antibodies.. Endocytobiology.

[18] Nogge G, Gerresheim A (1982). Experiments on the elimination of symbionts from the tsetse-Fly, *Glossina-Morsitans-Morsitans* (Diptera, *Glossinidae*), by antibiotics and lysozyme.. J Invertebr Pathol.

[19] O'Neill SL, Gooding RH, Aksoy S (1993). Phylogenetically distant symbiotic microorganisms reside in *Glossina* midgut and ovary tissues.. Med Vet Entomol.

[20] Cheng Q, Ruel TD, Zhou W, Moloo SK, Majiwa P (2000). Tissue distribution and prevalence of *Wolbachia* infections in tsetse flies, *Glossina* spp.. Med Vet Entomol.

[21] Pais R, Lohs C, Wu Y, Wang J, Aksoy S (2008). The obligate mutualist *Wigglesworthia glossinidia* influences reproduction, digestion, and immunity processes of its host, the tsetse fly.. Appl Environ Microbiol.

[22] Werren JH (1997). Biology of *Wolbachia*.. Annu Rev Entomol.

[23] Werren JH, Baldo L, Clark ME (2008). *Wolbachia*: master manipulators of invertebrate biology.. Nat Rev Microbiol.

[24] Saridaki A, Bourtzis K (2010). *Wolbachia*: more than just a bug in insects genitals.. Curr Opin Microbiol.

[25] Dobson SL, Fox C, Jiggins FM (2002). The effect of *Wolbachia*-induced cytoplasmic incompatibility on host population size in natural and manipulated systems.. Proc Biol Sci.

[26] Hoffman AA, Hercus M, Dagher H (1998). Population Dynamics of the *Wolbachia* infection causing cytoplasmic incompatibility in *Drosophila melanogaster*.. Genetics.

[27] Sinkins SP, Gould F (2006). Gene drive systems for insect disease vectors.. Nat Rev Genet.

[28] Rasgon J (2007). Population replacement strategies for controlling vector populations and the use of *Wolbachia pipientis* for genetic drive..

[29] Brelsfoard CL, Dobson SL (2009). *Wolbachia-*based strategies to control insect pests and disease vectors. Asia Pac. J. Mol. Biol.. Biotechnol.

[30] Rasgon JL (2008). Using predictive models to optimize *Wolbachia*-based strategies for vector-borne disease control.. Adv Exp Med Biol.

[31] Van Meer MMM, Witteveldt J, Stouthamer R (1999). Phylogeny of the arthropod endosymbiont *Wolbachia* based on the *wsp* gene.. Insect Mol Biol.

[32] Nogge G (1981). Significance of symbionts for the maintenance of an optimal nutritional state for successful reproduction in hematophagous arthropods.. Parasitology.

[33] Ballard JWO, Melvin RG (2007). Tetracycline treatment influences mitochondrial metabolism and mtDNA density two generations after treatment in *Drosophila*.. Insect Mol Biol.

[34] Dobson SL, Rattanadechakul W, Marsland EJ (2004). Fitness advantage and cytoplasmic incompatibility in *Wolbachia* single- and superinfected *Aedes albopictus*.. Heredity:.

[35] Dean M (2006). A *Wolbachia*-associated fitness benefit depends on genetic backgroun in *Drosophila simulans*.. Proc Biol Sci.

[36] Clark ME, Veneti Z, Bourtzis K, Karr TL (2002). The distribution and proliferation of the intracellular bacteria *Wolbachia* during spermatogenesis in *Drosophila*.. Mech Dev.

[37] Clark ME, Veneti Z, Bourtzis K, Karr TL (2003). *Wolbachia* distribution and cytoplasmic incompatibility during sperm development: the cyst as the basic cellular unit of CI expression.. Mech Dev.

[38] Clark ME, Bailey-Jourdain C, Ferree PM, England SJ, Sullivan W (2008). *Wolbachia* modification of sperm does not always require residence within developing sperm.. Heredity.

[39] Veneti Z, Clark ME, Zabalou S, Karr T, Savakis C (2003). Cytoplasmic incompatibility and sperm cyst infection in different *Drosophila*-*Wolbachia* association.. Genetics.

[40] Ferree PM, Frydman HM, Li JM, Cao J, Wieschaus E (2005). *Wolbachia* utilizes host microtubules and dynein for anterior localization in the *Drosophila* oocyte.. PloS Pathog.

[41] Serbus LR, Sullivan W (2007). A cellular basis for *Wolbachia* recruitment to the host germline.. PloS Pathog.

[42] Veneti Z, Clark ME, Karr TL, Savakis C, Bourtzis K (2004). Heads or tails: host-parasite interactions in the *Drosophila*-*Wolbachia* system.. Appl Environ Microbiol.

[43] Tram U, Fredrick K, Werren JH, Sullivan W (2006). Paternal chromosomal segregation during the frist mitotic division determines *Wolbachia*-induced cytoplasmic incompatibility phenotype.. J Cell Sci.

[44] Xi Z, Khoo CC, Dobson SL (2006). Interspecific transfer of *Wolbachia* into the mosquito disease vector *Aedes albopictus*.. Proc Biol Sci.

[45] Charlat S, Hurst GD, Mercot H (2003). Evolutionary consequences of *Wolbachia* infections.. Trends Genet.

[46] Dobson SL (2004). Evolution of *Wolbachia* cytoplasmic incompatibility types.. Evolution.

[47] Doudoumis V, Tsiamis G, Wamwiri F, Brelsfoard C, Alam U (in press) Detection and characterization of *Wolbachia* infections in laboratory and natural populations of different species of tsetse (genus *Glossina*)..

[48] Hoffmann AA, Turelli M, Harshman LG (1990). Factors affecting the distribution of cytoplasmic incompatibility in *Drosophila simulans*.. Genetics.

[49] Hoffmann A, Hercus M, Dagher H (1998). Population dynamics of the *Wolbachia* infection causing cytoplasmic incompatibility in *Drosophila melanogaster*.. Genetics.

[50] Weeks A, Turelli M, Harcombe W, Reynolds K, Hoffman AA (2007). From parasite to mutualist: rapid evolution of *Wolbachia* in natural populations of *Drosophila*.. PLoS Biol.

[51] Beard CB, O'Neill SL, Mason P, Mandelco L, Woese CR (1993). Genetic transformation and phylogeny of bacterial symbionts from tsetse.. Insect Mol Biol.

[52] Cheng Q, Aksoy S (1999). Tissue tropism, transmission and expression of foreign genes *in vivo* in midgut symbionts of tsetse flies.. Insect Mol Biol.

[53] Hu YJ, Aksoy S (2005). An antimicrobial peptide with trypanocidal activity characterized from *Glossina morsitans morsitans*.. Insect Biochem Mol Biol.

[54] Rio RV, Wu YN, Filardo G, Aksoy S (2006). Dynamics of multiple symbiont density regulation during host development: tsetse fly and its microbial flora.. Proc Biol Sci.

[55] Brelsfoard CL, Sechan Y, Dobson SL (2008). Interspecific hybridization yields strategy for South Pacific filariasis vector elimination.. PLoS Negl Trop Dis.

[56] Zabalou S, Riegler M, Theodorakopoulou M, Stauffer C, Savakis C (2004). *Wolbachia*-induced cytoplasmic incompatibility as a means for insect pest population control.. Proc Natl Acad Sci U S A.

[57] Laven H (1967). Eradication of *Culex pipiens fatigans* through cytoplasmic incompatibility.. Nature.

[58] Zabalou S, Apostolaki A, Livadaras I, Franz G, Robinson A (2009). Incompatible insect technique: incompatible males from a *Ceratitis capitata* (Diptera: *Tephritidae*) gentic sexing strain.. Entomol Exp Appl.

[59] Vreysen M, Saleh K, Lancelot R, Bouyer J (2011). Factory Tsetse flies must behave like wild flies: A prerequisite for the sterile insect technique.. PLoS Negl Trop Dis.

[60] McMeniman CJ, Lane RV, Cass BN, Fong AW, Sidhu M (2009). Stable introduction of a life-shortening *Wolbachia* infection into the mosquito *Aedes aegypti*.. Science.

[61] Xi Z, Khoo CCH, Dobson SL (2005). *Wolbachia* establishment and invasion in an *Aedes aegypti* laboratory population.. Science.

[62] Frydman HM, Li JM, Robson DN, Wieschaus E (2006). Somatic stem cell niche tropism in *Wolbachia*.. Nature.

[63] Ruang-Areerate T, Kittayapong P (2006). *Wolbachia* transinfection in *Aedes aegypti*: a potential gene driver of dengue vectors.. Proc Natl Acad Sci U S A.

[64] Turelli M, Hoffman A (1999). Microbe induced cytoplasmic incompatibility as a mechanism for introducing genes into arthropod populations.. Insect Mol Biol.

[65] Osborne SE, Leong YS, O'Neill SL, Johnson KN (2009). Variation in antiviral protection mediated by different *Wolbachia* strains in *Drosophila simulans*.. PLoS Pathog.

[66] Kambris Z, Blagborough A, Pinto S, Blagrove M, Godfray H (2010). *Wolbachia* stimulates immune gene expression and inhibits *plasmodium* development in *Anopheles gambiae*.. PLoS Pathog.

[67] Kambris Z, Cook PE, Phuc HK, Sinkins SP (2009). Immune activation by life-shortening *Wolbachia* and reduced filarial competence in mosquitoes.. Science.

[68] Moreira LA, Iturbe-Ormaetxe I, Jeffery JA, Lu G, Pyke AT (2009). A *Wolbachia* symbiont in *Aedes aegypti* limits infection with dengue, Chikungunya and *Plasmodium*.. Cell.

[69] Bian G, Xu Y, Lu P, Xie Y, Xi Z (2010). The endosymbiotic bacterium *Wolbachia* induces resistance to dengue virus in *Aedes aegypti*.. PLoS Pathog.

[70] Teixeira L, Ferreira A, Ashburner M (2008). The bacterial symbiont *Wolbachia* induces resistance to RNA viral infections in *Drosophila melanogaster*.. PLoS Biol.

[71] Moloo SK (1971). An artificial feeding technique for *Glossina*.. Parasitology.

[72] Anselme C, Vallier A, Balmand S, Fauvarque MO, Heddi A (2006). Host PGRP gene expression and bacterial release in endosymbiosis of the weevil *Sitophilus zeamais*.. Appl Environ Microbiol.

