# Supplementary figures and images for: Wolbachia Symbiont Infections Induce Strong Cytoplasmic Incompatibility in the Tsetse Fly Glossina morsitans
Source: PLoS Pathog. 2011 Dec 8;7(12):e1002415. doi: 10.1371/journal.ppat.1002415 (PMC3234226; doi:10.1371/journal.ppat.1002415)

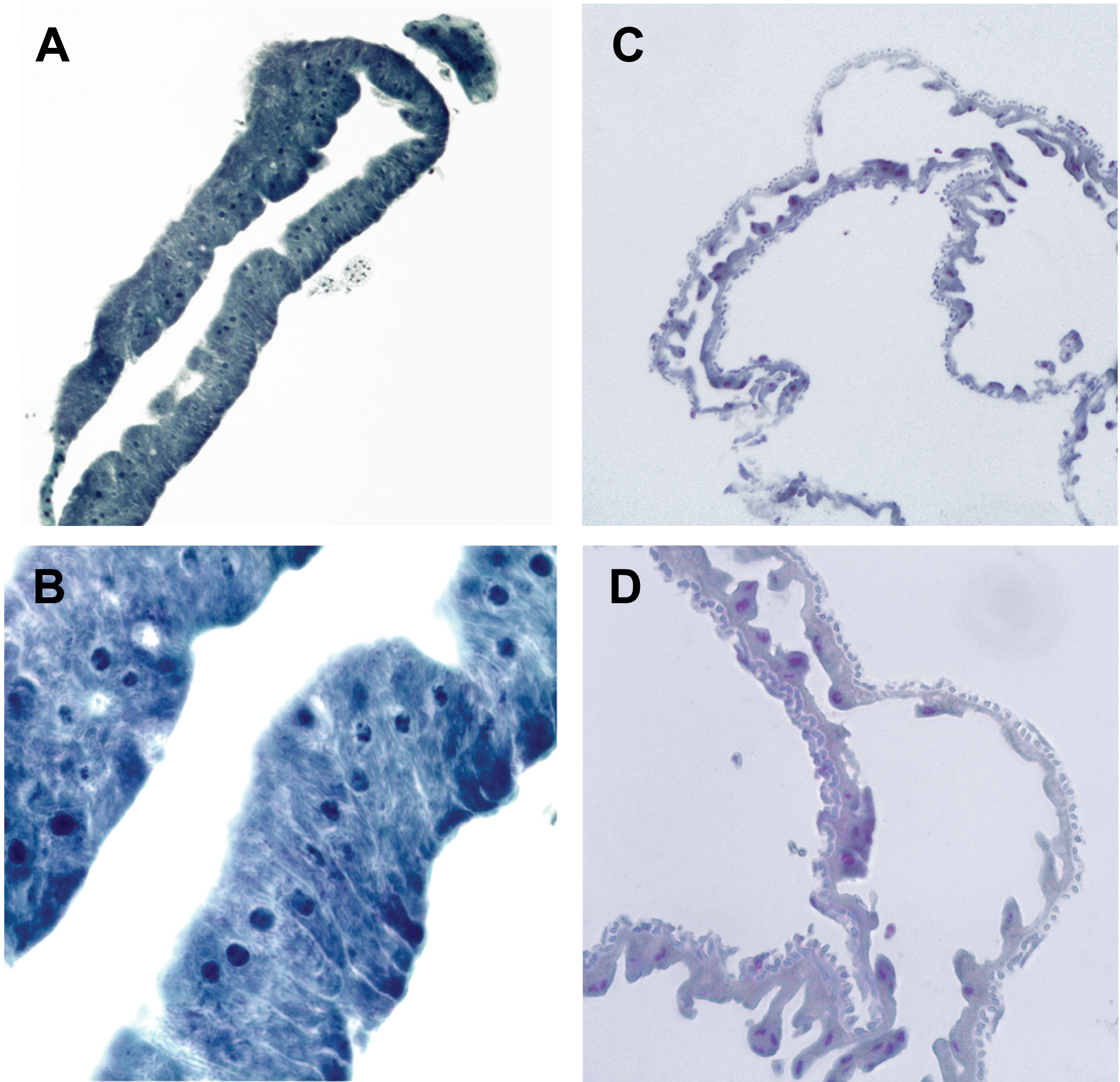

Supplement: Figure S1 — The effect of antibiotics on the bacteriome of G. m. morsitans. Images of bacteriome sections stained with Giemsa (A) bacteriome organ showing bacteriocytes harboring Wigglesworthia from a female maintained on normal bloodmeals, image taken at 10x magnification (B) bacterioctes taken 40 magnification from a female maintained on ampicillin supplemented diet. A normal bacteriome structure is retained on the ampicillin diet allowing for continued fertility of such females. (C and D) Bacteriome structure observed in the progeny of ampicillin receiving females (C) and tetracycline and yeast extract receiving females in (D). In these individuals, the bacteriocyctes lack Wigglesworthia and these females are reproductively sterile, images taken at 10x magnification. (TIF) [file ppat.1002415.s001.tif]
