# Supplementary material for: Wolbachia Symbiont Infections Induce Strong Cytoplasmic Incompatibility in the Tsetse Fly Glossina morsitans
Source: PLoS Pathog. 2011 Dec 8;7(12):e1002415. doi: 10.1371/journal.ppat.1002415 (PMC3234226; doi:10.1371/journal.ppat.1002415)
Supplement: Table S1 — Symbiont PCR primers. (PDF) [file ppat.1002415.s002.pdf]

Table S1. Symbiont PCR primers

	Gene	Primer Sequence
Sodalis	*exochitinase*	5'-CAAAGAAGTCATAGGTCATAAC-3'<br>5'-ACCGACTGGGGACAGTACGATG-3'
Wigglesworthia	Thic	5'-TGAAAACATTTGCAAAATTTG-3'<br>5'-GGTGTTACATAGCATAACAT-3'
Wolbachia	groEl	5'-GGTGAGCAGTTGCAAGAAGC-3'<br>5'-AGATCTTCCATCTTGATTCC-3'
Tsetse	*β-Tubulin*	5'-TAGTTCTCTTCAACTTCAGCCTCTT-3'<br>5'-TCGTTGACCATGTCTGGTGT-3'
