# Supplementary material for: Wolbachia Symbiont Infections Induce Strong Cytoplasmic Incompatibility in the Tsetse Fly Glossina morsitans
Source: PLoS Pathog. 2011 Dec 8;7(12):e1002415. doi: 10.1371/journal.ppat.1002415 (PMC3234226; doi:10.1371/journal.ppat.1002415)
Supplement: Table S2 — Larval deposition and pupal eclosion data for single cage crosses. In three separate experiments % larval deposition and % eclosion of the pupa deposited was determined. For each experiment, number of larval deposited for surviving females over two gonotrophic cycles and number of their pupae that hatched were recorded. Larval deposition was used as a measure of CI expression. To analyze for CI in replicate experiments of individual crosses, multiple Wilcoxon tests, with a Bonferroni correction were conducted to compare larval deposition rates. Wilcoxon tests, with a Bonferrroni correction were also conducted to compare pupal eclosion. Superscripted letters indicate significant differences, P<0.01. (PDF) [file ppat.1002415.s003.pdf]

**Table S2:** Larval deposition and pupal eclosion data for single cage crosses. In three separate experiments % larval deposition and % eclosion of the pupa deposited was determined.  For each experiment, number of larval deposited for surviving females over two gonotrophic cycles and number of their pupae that hatched were recorded. Larval deposition was used as a measure of CI expression. To analyze for CI in replicate experiments of individual crosses, multiple Wilcoxon tests, with a Bonferroni correction were conducted to compare larval deposition rates.  Wilcoxon tests, with a Bonferrroni correction were also conducted to compare pupal eclosion.  Superscripted letters indicate significant differences, $P < 0.01$.

| | **Experiment 1** | | | | **Experiment 2** | | | | **Experiment 3** | | | | **Mean of combined experiments** | | | |
|---|---|---|---|---|---|---|---|---|---|---|---|---|---|---|---|---|
| | **Larval deposition** | | **Pupal eclosion** | | **Larval deposition** | | **Pupal eclosion** | | **Larval deposition** | | **Pupal eclosion** | | **Larval deposition** | | **Pupal eclosion** | |
| | # Larva deposited/total females alive, % larval deposition | | # pupae eclosed/total pupae deposited, % pupal eclosion | | # Larva deposited/total females alive, % larval deposition | | # pupae eclosed/total pupae deposited, % pupal eclosion | | # Larva deposited/total females alive, % larval deposition | | # pupae eclosed/total pupae deposited, % pupal eclosion | | Mean % larval deposition ± SE | | Mean % pupal eclosion ± SE | |
| Cross type | 1st cycle | 2nd cycle | 1st cycle | 2nd cycle | 1st cycle | 2nd cycle | 1st cycle | 2nd cycle | 1st cycle | 2nd cycle | 1st cycle | 2nd cycle | 1st cycle | 2nd cycle | 1st cycle | 2nd cycle |
| ♀$Gmm^{Wt}$ x ♂$Gmm^{Wt}$ | 13/13, 100% | 7/8, 88% | 12/13, 92% | 6/7, 86% | 11/11, 100% | 3/3, 100% | 8/11, 73% | 3/3, 100% | 7/7, 100% | 7/7, 100% | 3/7, 43% | 0/7, 0% | 100 ± 0%[a] | 96.0 ± 4.0%[a] | 69.3 ± 14.3% | 62.0 ± 31.3% |
| ♀$Gmm^{Wt}$ x ♂$Gmm^{Apo}$ | 13/13, 100% | 10/10, 100% | 12/13, 92% | 10/10, 100% | 13/14, 93% | 5/5, 100% | 12/13, 92% | 2/5, 40% | 13/13, 100% | 5/5, 100% | 11/13, 85% | 4/5, 80% | 97.7 ± 2.3%[a] | 100 ± 0%[a] | 89.7 ± 2.3% | 73.3 ± 17.6% |
| ♀$Gmm^{Apo}$ x ♂$Gmm^{Apo}$ | 7/7, 100% | 6/6, 100% | 3/7, 43% | 6/6, 100% | 1/1, 100% | 1/1, 100% | 1/1, 100% | 1/1, 100% | 12/12, 100% | 5/5, 100% | 6/12, 50% | 1/5, 20% | 100 ± 0%[a] | 100 ± 0%[a] | 64.3 ± 17.9% | 73.3 ± 26.7% |
| ♀$Gmm^{Apo}$ x ♂$Gmm^{Wt}$ | 1/14, 7% | 0/12, 0% | 0/1, 0% | n/a | 0/2, 0% | 0/2, 0% | n/a | n/a | 1/17, 6% | 0/7, 0% | 1/1, 100% | n/a | 4.3 ± 2.2%[b] | 0 ± 0[b] | 50.0 ± 5.0% | n/a |
