# Supplementary material for: Wolbachia Symbiont Infections Induce Strong Cytoplasmic Incompatibility in the Tsetse Fly Glossina morsitans
Source: PLoS Pathog. 2011 Dec 8;7(12):e1002415. doi: 10.1371/journal.ppat.1002415 (PMC3234226; doi:10.1371/journal.ppat.1002415)
Supplement: Text S1 — Mathematical methods. (PDF) [file ppat.1002415.s004.pdf]

Mathematical methods

We describe the mathematical models used in analyzing the mating-cross data and extrapolating from these data to the population genetics of *Wolbachia* in a population of tsetse. A mathematical model have been developed and well studied for *Wolbachia*-induced cytoplasmic incompatibility in *Drosophila* [4]. This model assumes non-overlapping generations, which is particularly at odds with tsetse life history [5]. To overcome this, we constructed a continuous-time model for the time evolution of the number of *Wolbachia*-infected and uninfected tsetse in a population using differential equations. We first discuss this new population-genetic model and its analysis, showing that it has the same threshold phenomenon as the earlier discrete-time model. Then we describe the Bayesian Markov chain Monte Carlo method used to estimate model parameters and derived quantities from the data.

1 *Wolbachia* Population Genetics

Let $N_p(t)$ and $N_n(t)$ be the numbers of *Wolbachia*-infected and *Wolbachia*-uninfected tsetse, respectively, at time t and $N(t) = N_p(t) + N_n(t)$. Then we have

$$\begin{aligned}\frac{\mathrm{d}N_n}{\mathrm{d}t} &= rf\left[\frac{N_n}{N} + (1-s_h)\frac{N_p}{N}\right]N_n \\ &\quad + (1+s_r)r(1+s_f)f\mu\left[\frac{N_n}{N} + (1-s_h)\frac{N_p}{N}\right]N_p - dN_n, \qquad (1a)\\ \frac{\mathrm{d}N_p}{\mathrm{d}t} &= (1+s_r)r(1+s_f)f(1-\mu)\left(\frac{N_n}{N} + \frac{N_p}{N}\right)N_p \\ &\quad - d(1+s_d)N_p, \qquad (1b)\end{aligned}$$

where r is the offspring-production rate (that is, frequency of gonotrophic cycles) of *Wolbachia*-uninfected tsetse, f is the probability of an *Wolbachia*-uninfected tsetse successfully producing an offspring during a given gonotrophic cycle (i.e. the probability of a fertilized egg maturing to a larva), d is the death rate of *Wolbachia*-uninfected tsetse, and μ is the proportion of *Wolbachia*-uninfected eggs of *Wolbachia*-infected mothers. For *Wolbachia*-infected tsetse, $1 + s_r$ is the relative offspring-production rate, $1 + s_f$ is the relative reproduction success, s_h is the proportion of fertilization of *Wolbachia*-uninfected eggs by

	Number of Offspring	
**♀** × **♂**	⚥	$\mu f(1+s_f)(1-s_h)$
	**⚥**	$(1-\mu)f(1+s_f)$
**♀** × ♂	⚥	$\mu f(1+s_f)$
	**⚥**	$(1-\mu)f(1+s_f)$
♀ × ♂	⚥	f
♀ × **♂**	⚥	$f(1-s_h)$

Table 1: Matings and offspring per offspring cycle. Open circles are *Wolbachia* uninfected and closed are *Wolbachia* infected. For the offspring, the expression is the relative number of offspring, given that such a mating occurs.

Wolbachia-infected sperm that are not viable ($s_h = 1$ means no such fertilizations produce offspring), and $1 + s_d$ is the relative death rate (Table 1). In equation (1a), the first term is births of *Wolbachia*-uninfected offspring from *Wolbachia*-infected mothers, while the second term is births of *Wolbachia*-uninfected offspring from *Wolbachia*-uninfected mothers. *Wolbachia*-uninfected eggs can be fertilized by *Wolbachia*-uninfected males (proportion N_n/N of the population) successfully or by *Wolbachia*-infected males (proportion N_p/N of the population) with probability of success $1 - s_h$. The final term in equation (1a) represents death. For equation (1b), the first term is births of *Wolbachia*-infected offspring, which requires that the mother be *Wolbachia*-infected (N_p), that she produces eggs at rate $(1 + s_r)r$, those eggs mature to larva with probability $(1 + s_f)f$, that proportion $(1 - \mu)$ of those eggs are *Wolbachia* infected, and that she mates with either a *Wolbachia*-infected male (proportion N_p/N of the population) or a *Wolbachia*-uninfected male (proportion N_n/N of the population). The second term in equation (1b) represents death.

Using $N = N_n + N_p$ simplifies the model to

$$\begin{aligned}\frac{\mathrm{d}N_n}{\mathrm{d}t} &= rf\left(1 - s_h\frac{N_p}{N}\right)\left[N_n + (1+s_r)(1+s_f)\mu N_p\right] - dN_n,\\ \frac{\mathrm{d}N_p}{\mathrm{d}t} &= (1+s_r)r(1+s_f)f(1-\mu)N_p - d(1+s_d)N_p.\end{aligned} \tag{2}$$

Letting $p(t) = N_p(t)/N(t)$ gives a single differential equation for the time evolution of the proportion of the population infected with *Wolbachia*

$$\begin{aligned}\frac{\mathrm{d}p}{\mathrm{d}t} = &-\left(\{rf[1-(1+s_r)(1+s_f)(1-\mu)] + s_d d\}\right.\\ &- \{rf[1-(1+s_r)(1+s_f)+s_h] + s_d d\}\,p\\ &\left.+ rfs_h\left[1-\mu(1+s_r)(1+s_f)\right]p^2\right)p.\end{aligned} \tag{3}$$

1.1 Model Analysis

We will show that model (3) can have a threshold, above which *Wolbachia* prevalence increases in time and below which prevalence decreases in time, however this threshold is not present for all parameter values, exactly as for the model with non-overlapping generations [4]. Importantly, the presence of such a threshold indicates that there is some minimum effort needed for successful population transformation using *Wolbachia* as a driver [6, 2, 7].

Letting $\tau = rft$ and $\delta = s_d d/r$ gives the non-dimensional model

$$\begin{aligned}\frac{\mathrm{d}p}{\mathrm{d}\tau} = &- \{[1-(1+s_r)(1+s_f)(1-\mu)+\delta] \\ &- [1-(1+s_r)(1+s_f)+s_h+\delta]\,p \\ &+ s_h\,[1-\mu(1+s_r)(1+s_f)]\,p^2\}\,p,\end{aligned} \tag{4}$$

with the basic biological conditions on the parameters

$$\begin{aligned} &r>0, \quad d>0, \quad 0<f\le 1, \quad 0\le\mu<1, \\ &0<s_h\le 1, \quad s_r>-1, \quad s_d>-1, \quad -1<s_f<\frac{1-f}{f}. \end{aligned} \tag{5}$$

From model (4),

$$\left.\frac{\mathrm{d}p}{\mathrm{d}\tau}\right|_{p=0} = 0 \quad \text{and} \quad \left.\frac{\mathrm{d}p}{\mathrm{d}\tau}\right|_{p=1} = -(1+s_r)(1+s_f)\mu(1-s_h) \le 0, \tag{6}$$

show that the interval $0 \le p \le 1$ is forward invariant, meaning that if initially $0 \le p(0) \le 1$, then $0 \le p(t) \le 1$ for all $t \ge 0$. This, in addition to fact that the model is a cubic polynomial, greatly restricts the possible phase space. Obviously, $p_0 = 0$ is an equilibrium and, when $\mu = 0$ (perfect *Wolbachia* transmission) or $s_h = 1$ (perfect CI), $p = 1$ is also an equilibrium. We consider only the general case of imperfect transmission ($\mu > 0$) and imperfect CI ($s_h < 1$) in detail, ignoring the special cases with perfect CI or perfect transmission. Briefly, in these special cases, for some parameter values, $p = 1$ is an unstable equilibrium, so that *Wolbachia* can persist in the population at 100% prevalence until the introduction of any *Wolbachia*-negative tsetse, at which point the *Wolbachia* prevalence tends to some lower value, perhaps even $p_0 = 0$. In these case, at $p = 1$, *Wolbachia*-negative tsetse have higher fitness than *Wolbachia*-positive tsetse, but are not present in the population: if they are introduced, they replace at least a portion of the *Wolbachia*-positive population. This phenomenon of an unstable equilibrium with high *Wolbachia* prevalence does not occur in the general case because with imperfect transmission and imperfect CI, some number of *Wolbachia*-negative are always present, being produced as offspring of *Wolbachia*-positive mothers.

In general, there is a state with no *Wolbachia* in the population(p_0); a state where *Wolbachia* has reached fixation in the population (p_F); and there may be a threshold state (p_T), above which *Wolbachia* goes to fixation and below which *Wolbachia* goes to extinction. There are three possibilities for the general case of imperfect transmission and imperfect CI:

1. *Wolbachia* can never persist in the population. *Wolbachia*-positive tsetse have lower fitness at all levels of prevalence. (There is one equilibrium, $p_F = p_0 = 0$, and it is stable.)

2. The presence of any amount of *Wolbachia* leads to fixation in the population (i.e. there is no release threshold). *Wolbachia*-positive tsetse have higher fitness at all levels of prevalence. The fixation prevalence is less than 100% due to some failure of transmission. (There are two equilibria, $p_T = p_0 = 0$ and p_F, with $0 = p_0 < p_F < 1$, p_0 unstable, and p_F stable.)

3. There is a release threshold (p_T) so that if *Wolbachia* prevalence is above the threshold, it will go to fixation, and if *Wolbachia* prevalence is below the threshold, it will be driven out of the population. *Wolbachia*-positive tsetse have lower fitness at levels of prevalence below the threshold and higher fitness at levels of prevalence above the threshold. The fixation prevalence is less than 100% due to some failure of transmission. (There are three equilibria, $p_0 = 0$, p_T, and p_F, with $0 = p_0 < p_T < p_F < 1$, p_0 stable, p_T unstable, and p_F stable.)

1.2 Time to fixation

A quantity of interest is the amount of time it takes for *Wolbachia* to go from an initial introduction in a small number of tsetse to fixation in the tsetse population. To get a more accurate estimate of this time in the presence of the unique reproductive biology of tsetse, we extended model (2) to separate the female population into females before the deposition of their first pupal offspring and females after this deposition because the time from a female being deposited as a pupa to the deposition of her first pupal offspring is significantly longer than the time between her subsequent pupal depositions.

Let $N_{p0}(t)$ be the number of *Wolbachia*-infected female tsetse before the deposition of their first pupal offspring at time t, and $N_{p1}(t)$ be the number of *Wolbachia*-infected female tsetse after the deposition of their first pupal offspring at time t. Let $N_{n0}(t)$ and $N_{n1}(t)$ be similarly defined for *Wolbachia*-uninfected female tsetse. For *Wolbachia*-uninfected females, let r_0 be the production rate for the first offspring and r_1 be the production rate for subsequent offspring (i.e. $1/r_0$ and $1/r_1$ are the mean times to first offspring and to subsequent offspring, the duration of the gonotrophic cycle). For *Wolbachia*-infected females, let $(1+s_{r_0})r_0$ be the production rate for the first offspring and $(1+s_{r_1})r_1$ be the production rate

for subsequent offspring females, so that s_{r_0} and s_{r_1} are the relative offspring-production benefits for *Wolbachia* infection. Then we have

$$\begin{aligned}
\frac{\mathrm{d}N_{n0}}{\mathrm{d}t} &= f\left(1 - s_h\frac{N_p}{N}\right)\{r_0N_{n0} + r_1N_{n1} + (1+s_f)\mu\left[(1+s_{r_0})r_0N_{p0} + (1+s_{r_1})r_1N_{p1}\right]\} \\
&\qquad - r_0N_{n0} - dN_{n0}, \\
\frac{\mathrm{d}N_{n1}}{\mathrm{d}t} &= r_0N_{n0} - dN_{n1}, \\
\frac{\mathrm{d}N_{p0}}{\mathrm{d}t} &= (1+s_f)f(1-\mu)\left[(1+s_{r_0})r_0N_{p0} + (1+s_{r_1})r_1N_{p1}\right] \\
&\qquad - (1+s_{r_0})r_0N_{p0} - d(1+s_d)N_{p0}, \\
\frac{\mathrm{d}N_{p1}}{\mathrm{d}t} &= (1+s_{r_0})r_0N_{p0} - d(1+s_d)N_{p1},
\end{aligned} \tag{7}$$

with $N_n = N_{n0} + N_{n1}$, $N_p = N_{p0} + N_{p1}$, and $N = N_n + N_p$. The $-r_0N_{n0}$ term in the first equation and the $r_0N_{n_0}$ term in the second equation are the transition of a *Wolbachia*-uninfected mother's status at her first deposition; likewise the terms $\mp(1+s_{r_0})r_0N_{p0}$ in the third and fourth equations are the same transition for *Wolbachia*-infected mothers. Note that in the event of the failure to produce a first offspring, the female still moves into the next stage class.

In a population free of *Wolbachia* ($N_{p0} = N_{p1} = 0$), the model becomes linear, giving the population growth rate

$$\lambda = \frac{\sqrt{r_0^2(1-f)^2 + 4r_0r_1f} - r_0(1-f) - 2d}{2} \tag{8}$$

and the stable stage distribution

$$\begin{aligned}
\frac{N_{n0}}{N} &= N_{n0}^* = \frac{2fr_1}{2fr_1 + (1-f)r_0 + \sqrt{r_0^2(1-f)^2 + 4r_0r_1f}}, \\
\frac{N_{n1}}{N} &= N_{n1}^* = \frac{(1-f)r_0 + \sqrt{r_0^2(1-f)^2 + 4r_0r_1f}}{2fr_1 + (1-f)r_0 + \sqrt{r_0^2(1-f)^2 + 4r_0r_1f}}.
\end{aligned} \tag{9}$$

(See e.g. Caswell [1].)

To facilitate numerical solution, this model was converted from numbers of tsetse in each class to the proportions

$$n_{n0} = \frac{N_{n0}}{N}, \quad n_{n1} = \frac{N_{n1}}{N}, \quad n_{p0} = \frac{N_{p0}}{N}, \quad n_{p1} = \frac{N_{p1}}{N}. \tag{10}$$

The resulting model equations are

$$
\begin{aligned}
\frac{\mathrm{d}n_{n0}}{\mathrm{d}t} &= f(1 - s_h n_p)\left\{r_0 n_{n0} + r_1 n_{n1} + (1 + s_f)\mu\left[(1 + s_{r_0})r_0 n_{p0} + (1 + s_{r_1})r_1 n_{p1}\right]\right\} \\
&\quad - r_0 n_{n0} - d n_{n0} - n_{n0}\frac{1}{N}\frac{\mathrm{d}N}{\mathrm{d}t}, \\
\frac{\mathrm{d}n_{n1}}{\mathrm{d}t} &= r_0 n_{n0} - d n_{n1} - n_{n1}\frac{1}{N}\frac{\mathrm{d}N}{\mathrm{d}t}, \\
\frac{\mathrm{d}n_{p0}}{\mathrm{d}t} &= (1 + s_f)f(1 - \mu)\left[(1 + s_{r_0})r_0 n_{p0} + (1 + s_{r_1})r_1 n_{p1}\right] \\
&\quad - (1 + s_{r_0})r_0 n_{p0} - d(1 + s_d)n_{p0} - n_{p0}\frac{1}{N}\frac{\mathrm{d}N}{\mathrm{d}t}, \\
\frac{\mathrm{d}n_{p1}}{\mathrm{d}t} &= (1 + s_{r_0})r_0 n_{p0} - d(1 + s_d)n_{p1} - n_{p1}\frac{1}{N}\frac{\mathrm{d}N}{\mathrm{d}t},
\end{aligned}
\tag{11}
$$

where

$$
\begin{aligned}
\frac{1}{N}\frac{\mathrm{d}N}{\mathrm{d}t} = f\,&\{(1 - s_h n_p)(r_0 n_{n0} + r_1 n_{n1}) \\
&+ (1 + s_f)(1 - \mu s_h n_p)\left[(1 + s_{r_0})r_0 n_{p0} + (1 + s_{r_1})r_1 n_{p1}\right]\} \\
&- d(1 + s_d n_p).
\end{aligned}
\tag{12}
$$

The death rate, d, was chosen so that a *Wolbachia*-free population has a stable size (i.e. the growth rate $\lambda = 0$) and the relative mortality cost of *Wolbachia* infection was assumed to be $s_d = 0$. For *Wolbachia*-uninfected tsetse, we assumed a mean of 60 days between the deposition of a female pupa and that female depositing her first pupa ($r_0 = 1/60\ \mathrm{d}^{-1}$) and then 10-day intervals between her subsequent pupal depositions ($r_1 = 1/10\ \mathrm{d}^{-1}$). For *Wolbachia*-infected tsetse, we assumed 50 days to first deposition (so $(1 + s_{r_0})r_0 = 1/50\ \mathrm{d}^{-1} \implies s_{r_0} = 0.2$) and 10 days between subsequent depositions (so $(1 + s_{r_1})r_1 = 1/10\ \mathrm{d}^{-1} \implies s_{r_1} = 0$). Sensitivity analysis was performed on the relative costs s_{r_0}, s_{r_1}, and s_d (Figures 5–7).

2 Bayesian Markov Chain Monte Carlo Parameter Estimation

Following Gelman et al. [3], the Bayesian Markov chain Monte Carlo (MCMC) method was implemented using the Metropolis–Hastings algorithm, with a multi-dimensional normal distribution for the jumps. Maximum-likelihood estimates of the parameters were found first, using standard numerical minimization and the covariance approximated from the Hessian matrix. From these maximum-likelihood estimates, importance resampling was done to get 10 sets of 1000 samples each to start the MCMC routine. The MCMC was run with 10 parallel sequences to derive the stopping criterion based on the variances within and between sequences.

From the mating-cross data, for each cross let N be the number of females in the experiment and let P be the number of pupae deposited, with subscript fm denoting the female and male's type, respectively. The three types of tsetse are wild type (*Gmm*^{Wt} in the main text), denoted here by W, harboring *Wolbachia*, *Wigglesworthia*, & *Sodalis*; ampicillin treated (*Gmm*$^{Wig-}$), denoted by A, cleared of *Wigglesworthia*, but harboring *Wolbachia* & *Sodalis*; and tetracycline treated (*Gmm*^{Apo}), denoted by T, cleared of *Wolbachia*, *Wigglesworthia*, & *Sodalis*.

Assuming that each number of offspring is a binomial random variable gives the simple probability models for the data

$$\begin{aligned} P_{WW} &\sim \mathrm{B}\left(N_{WW}, q_{WW}\right), \\ P_{WT} &\sim \mathrm{B}\left(N_{WT}, q_{WT}\right), \\ P_{TW} &\sim \mathrm{B}\left(N_{TW}, q_{TW}\right), \\ P_{TT} &\sim \mathrm{B}\left(N_{TT}, q_{TT}\right), \\ P_{AA} &\sim \mathrm{B}\left(N_{AA}, q_{AA}\right), \end{aligned} \tag{13}$$

where $\mathrm{B}(N, p)$ is the standard binomial random variable for N Bernoulli trials with individual success probability q. Then the likelihood function for each of the models is simply that of the binomial distribution,

$$L(f, s_f, s_h, \mu | P) = \binom{N}{P} q^P (1-q)^{N-P}, \tag{14}$$

with the appropriate N, P, and q. The probabilities of pupal deposition for each mating cross are

$$\begin{aligned} q_{WW} &= f_W(1-\mu s_h), \\ q_{WT} &= f_W, \\ q_{TW} &= f_T(1-s_h), \\ q_{TT} &= f_T, \\ q_{AA} &= f_A(1-\mu s_h). \end{aligned} \tag{15}$$

The reproduction successes are

$$\begin{aligned} f_A &= f_T(1+s_{f,\mathrm{Wol}}), \\ f_W &= f_T(1+s_{f,\mathrm{Wol}})(1+s_{f,\mathrm{Wig}}), \end{aligned} \tag{16}$$

due to the relative reproduction-success benefits of the mother harboring *Wolbachia* ($s_{f,\mathrm{Wol}}$) and *Wigglesworthia* ($s_{f,\mathrm{Wig}}$). (Because ampicillin only cleared *Wigglesworthia* and tetracycline cleared *Wolbachia*, *Wigglesworthia*, & *Sodalis*, we were unable to estimate the impact of each bacteria separately. Here we are assuming that the difference between

ampicillin-treated and tetracycline-treated tsetse is entirely due to the absence of *Wolbachia*, and not to the absence *Sodalis*.)

Each of the parameters f_W, f_T, f_A, s_h, and μ are a proportion, so we use uninformative priors of uniform distributions on $(0, 1)$ for the parameters. The parameters were then transformed using the logit function

$$\operatorname{logit}(x) = \log(x) - \log(1 - x), \tag{17}$$

transforming them from the interval $(0, 1)$ to $(-\infty, +\infty)$.

In addition to estimating the parameters, quantities derived from those parameters were also estimated for each sample set of parameters.

- Reproduction-success benefits were calculated as

  $$s_{f,\mathrm{Wol}} = f_A/f_T - 1 \qquad \text{and} \qquad s_{f,\mathrm{Wig}} = f_W/f_A - 1. \tag{18}$$

- Fixation prevalence (p_F) was calculated as an equilibrium of population-genetics model (11). For each sample set of parameters, a nonlinear root finder was used to find the model equilibrium for *Wolbachia* fixation. The fixation prevalence was then calculated as $p_F = (N_{p0} + N_{p1})/N$. For this comparison, *Wolbachia*-negative tsetse were assumed to have reproduction success

  $$f = f_T(1 + s_{f,\mathrm{Wig}}) \tag{19}$$

  (i.e. the reproduction success with *Wigglesworthia* & *Sodalis*), while *Wolbachia*-positive tsetse had reproduction success

  $$f_W = f(1 + s_{f,\mathrm{Wol}}) \tag{20}$$

  (with *Wolbachia*, *Wigglesworthia*, & *Sodalis*).

- The release threshold (q_T), defined as the number of newly-emerged *Wolbachia*-positive tsetse (N_{p0}) that must be released to achieve eventual fixation of *Wolbachia*. This release threshold is relative to the size of the population, so that, for example, $q_T = 0.01$ indicates that release of the size of 1% of the population.

  For a given value of q, the initial condition

  $$n_{n0}(0) = N^*_{n0}, \quad n_{n1}(0) = N^*_{n1}, \quad n_{p0}(0) = q, \quad n_{p1}(0) = 0. \tag{21}$$

  was used, where N^*_{n0} and N^*_{n1} are the stable stage distribution for a *Wolbachia*-free population given in (9). The population-genetics model was solved numerically for 10 years. At the end of 10 years, for large values of q the *Wolbachia* is near fixation, while for small values of q the *Wolbachia* is near extinction. A bisection algorithm was used to find the critical value of q.

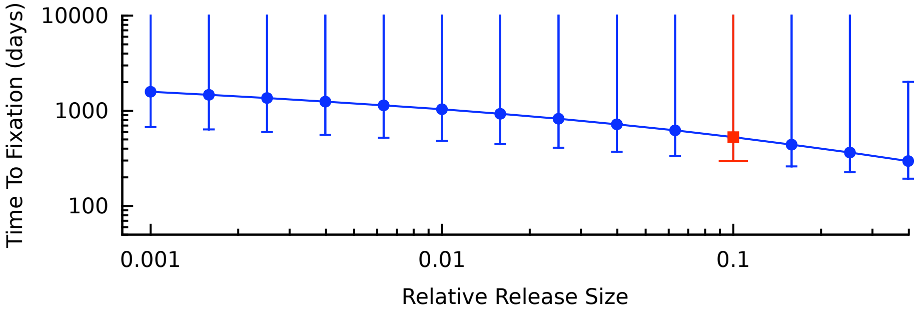

Figure 1: Time to fixation (τ) vs. release size (q). The red box and bars show the median and 95% credible interval for the baseline parameter values, while the blue circles and bars show the median 95% credible interval as the parameter is varied.

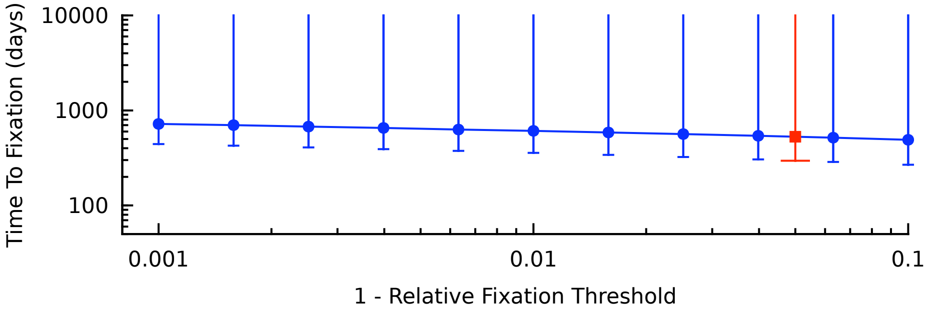

Figure 2: Time to fixation (τ) vs. relative fixation threshold (T). Note that here the x-axis is $1 - T$. The red box and bars show the median and 95% credible interval for the baseline parameter values, while the blue circles and bars show the median 95% credible interval as the parameter is varied.

- The time to fixation (τ) was calculated by numerically solving the population-genetics model from a small initial release of *Wolbachia*-infected tsetse ($q = 0.1$) until the population reached 95% (i.e. $T = 0.95$) of its fixation prevalence, p_F. Figures 1 and 2 show the sensitivity of time to fixation to changes in the initial-release size q and relative fixation threshold T used. These were generated by calculating the time to fixation for all of the sample parameter sets generated by the MCMC routine over ranges of q and T.

2.1 Sensitivity Analysis

Sensitivity analysis of fixation prevalence (p_F), release threshold (q_T), and time to fixation (τ) to various model parameters were also performed (Figures 3–12). Only one parameter was varied at a time from the baseline parameter values of $q = 0.1$ for release size; $T = 0.95$ for the relative fixation threshold; $r_0 = 1/60\ \mathrm{days}^{-1}$ for the rate of first deposition; $r_1 = 1/10\ \mathrm{days}^{-1}$ for the rate of subsequent depositions; $s_{r_0} = 0.2$ for the relative cost to rate of first deposition; $s_{r_1} = 0$ for the relative cost to rate of subsequent depositions; $s_d = 0$ for the relative mortality cost; and s_h, μ, f, $s_{f,\mathrm{Wol}}$, and $s_{f,\mathrm{Wig}}$ from the MCMC samples.

- Rate of first deposition (r_0), rate of subsequent depositions (r_1), relative benefit to rate of first deposition (s_{r_0}), relative benefit to rate of subsequent depositions (s_{r_1}), and relative mortality cost (s_d) were not estimated from the experimental data. For ranges of each of these parameters calculated, the model outputs were calculated using all of the sample parameter sets from the MCMC routine (Figures 3–7).

- Proportion incompatible crosses that fail (s_h), transmission failure (μ), and reproduction success of wild-type tsetse (f_W) were estimated from the data. To generate sensitivity to these parameters, the above model outputs were calculated with fixed values of one of these parameters and using the MCMC sample parameter sets for the other estimated parameters (Figures 8–10).

- The relative benefits to reproduction success for *Wolbachia* ($s_{f,\mathrm{Wol}}$) and for *Wigglesworthia* ($s_{f,\mathrm{Wig}}$) were estimated indirectly from the data by equations (18), where f_W, f_T, and f_A were directly estimated from data. To estimate the sensitivity, $s_{f,\mathrm{Wol}}$ values were chosen in a range and for each of those values, f_T was set to $f_A/(1 + s_{f,\mathrm{Wol}})$ and the MCMC parameter sets were used for the other parameters. Likewise, $s_{f,\mathrm{Wig}}$ values were chosen in a range and for each of those values, f_A was set to $f_W/(1 + s_{f,\mathrm{Wig}})$ and the MCMC parameter sets were used for the other parameters (Figures 11 & 12).

The model shows strong sensitivity to transmission failure (μ) and relative reproductive-success benefit to *Wolbachia* infection ($s_{f,\mathrm{Wol}}$), with some MCMC samples showing *Wolbachia* is unsustainable altogether for $\mu \geq 0.3$ or $s_{f,\mathrm{Wol}} \leq -0.1$ (Figures 9 & 11). The model shows weaker sensitivity to large negative values of relative benefit to rate of subsequent depositions (s_{r_1}), large values of relative cost to mortality (s_d), large values of proportion of incompatible crosses that fail (s_h), and large values of relative reproductive-success benefit to *Wigglesworthia* infection (Figures 6–8 & 12). There was weak sensitivity to rate of first deposition (r_0), rate of subsequent depositions (r_1), relative benefit to rate of first deposition (s_{r_0}), and wild-type reproduction success (f_W) (Figures 3–5 & 10).

Local sensitivity and elasticity analysis of fixation prevalence and time to fixation at the baseline parameter values was also performed by varying the non-zero parameters

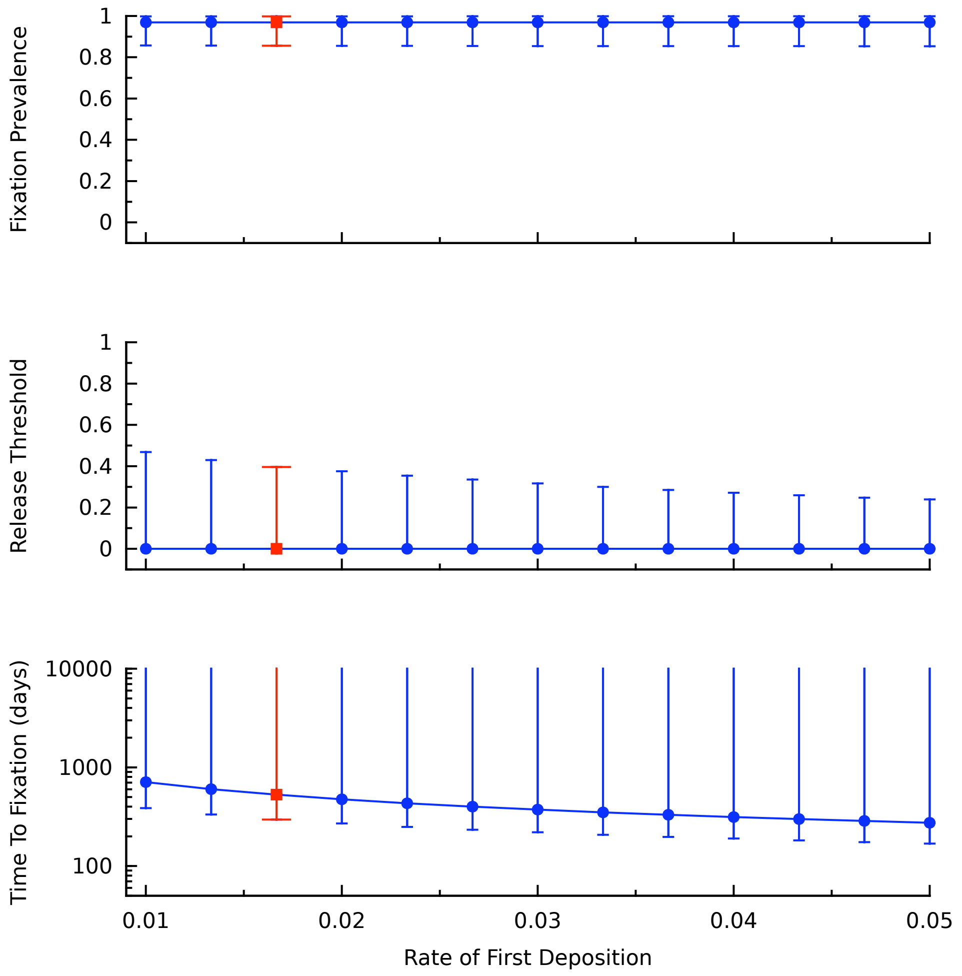

Figure 3: Fixation prevalence (p_F), release threshold (q_T) and time to fixation (τ) vs. rate of first deposition (r_0). The red box and bars show the median and 95% credible interval for the baseline parameter values, while the blue circles and bars show the median 95% credible interval as the parameter is varied.

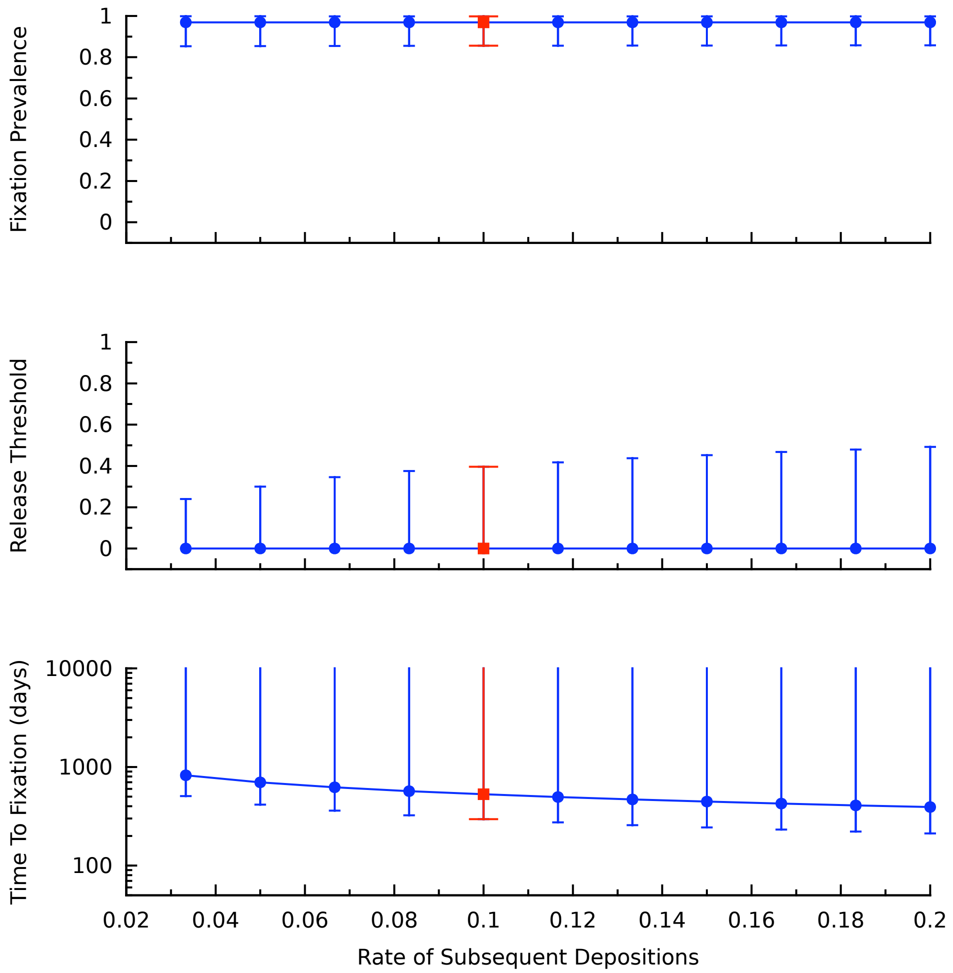

Figure 4: Fixation prevalence (p_F), release threshold (q_T) and time to fixation (τ) vs. rate of subsequent depositions (r_1). The red box and bars show the median and 95% credible interval for the baseline parameter values, while the blue circles and bars show the median 95% credible interval as the parameter is varied.

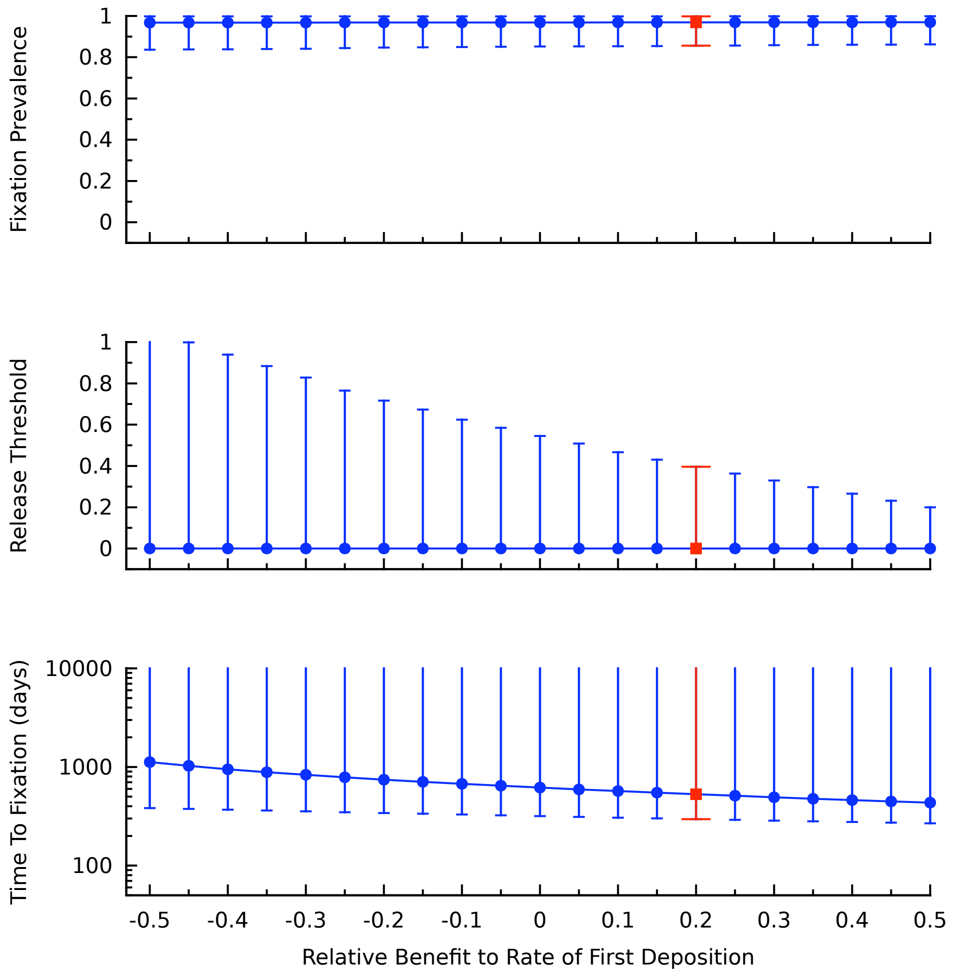

Figure 5: Fixation prevalence (p_F), release threshold (q_T) and time to fixation (τ) vs. relative benefit to rate of first deposition of *Wolbachia* infection (s_{r_0}). The red box and bars show the median and 95% credible interval for the baseline parameter values, while the blue circles and bars show the median 95% credible interval as the parameter is varied.

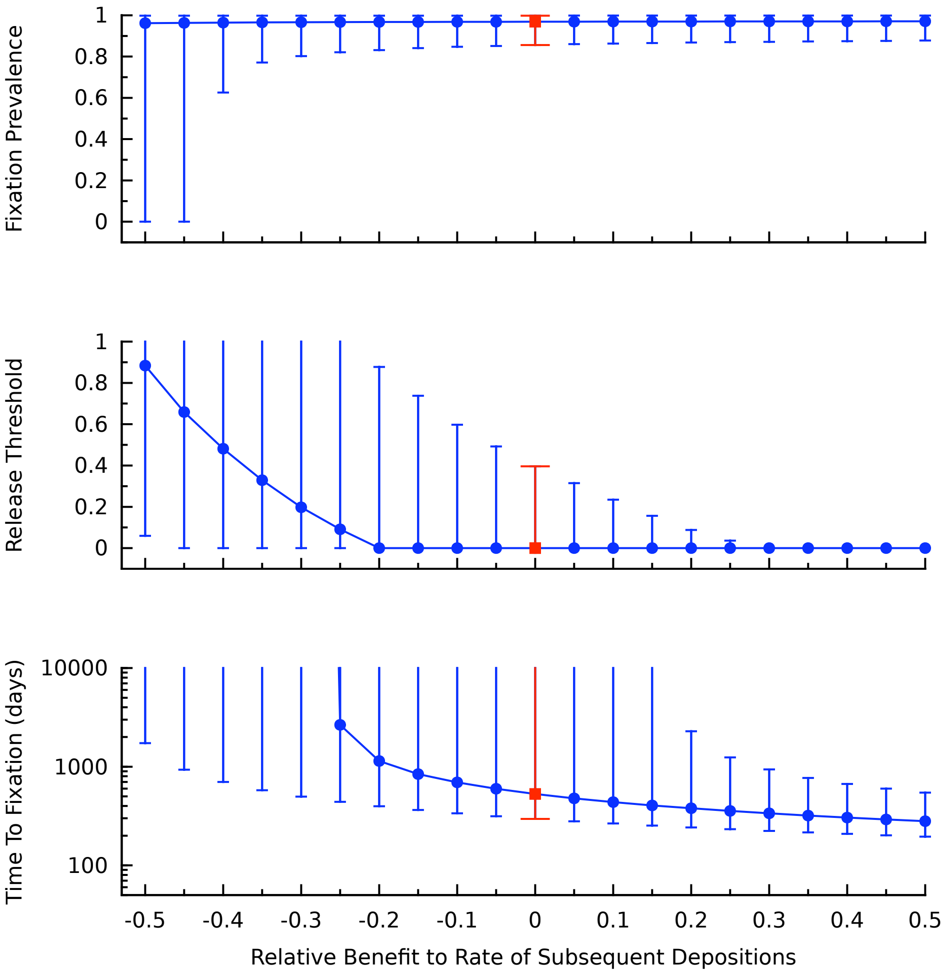

Figure 6: Fixation prevalence (p_F), release threshold (q_T) and time to fixation (τ) vs. relative benefit to rate of subsequent depositions of *Wolbachia* infection (s_{r_1}). The red box and bars show the median and 95% credible interval for the baseline parameter values, while the blue circles and bars show the median 95% credible interval as the parameter is varied.

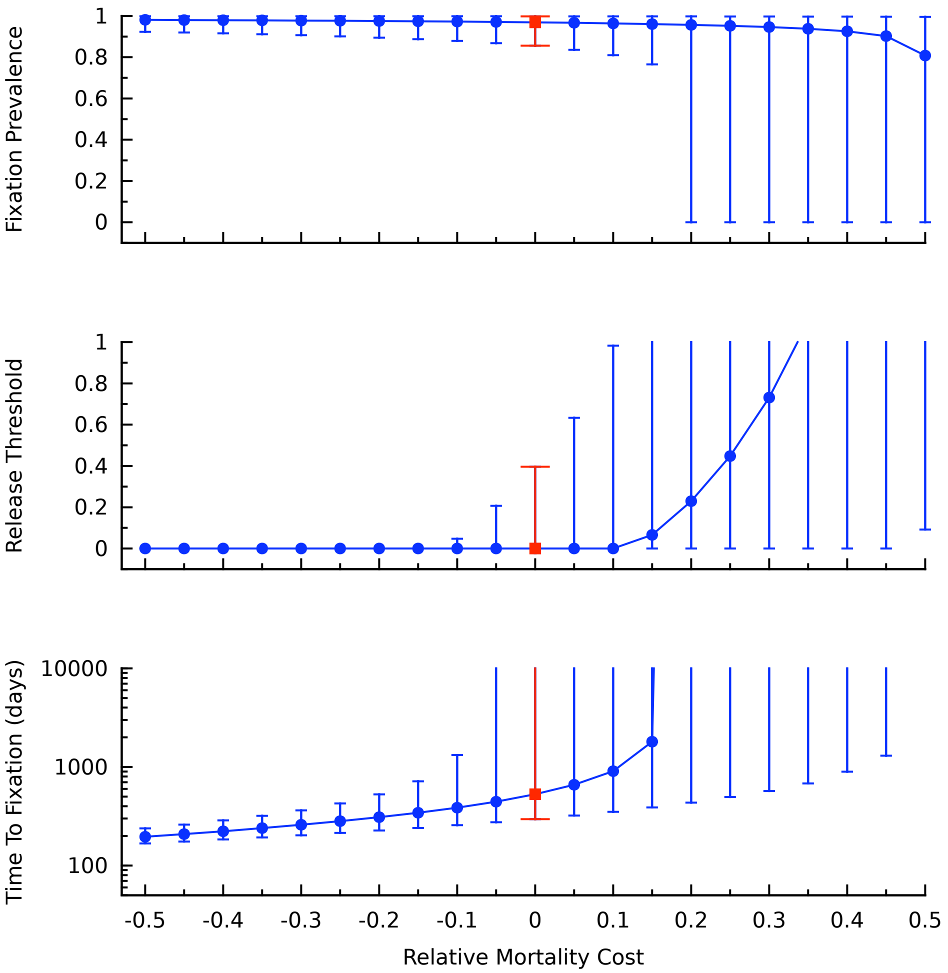

Figure 7: Fixation prevalence (p_F), release threshold (q_T) and time to fixation (τ) vs. relative mortality cost of *Wolbachia* infection (s_d). The red box and bars show the median and 95% credible interval for the baseline parameter values, while the blue circles and bars show the median 95% credible interval as the parameter is varied.

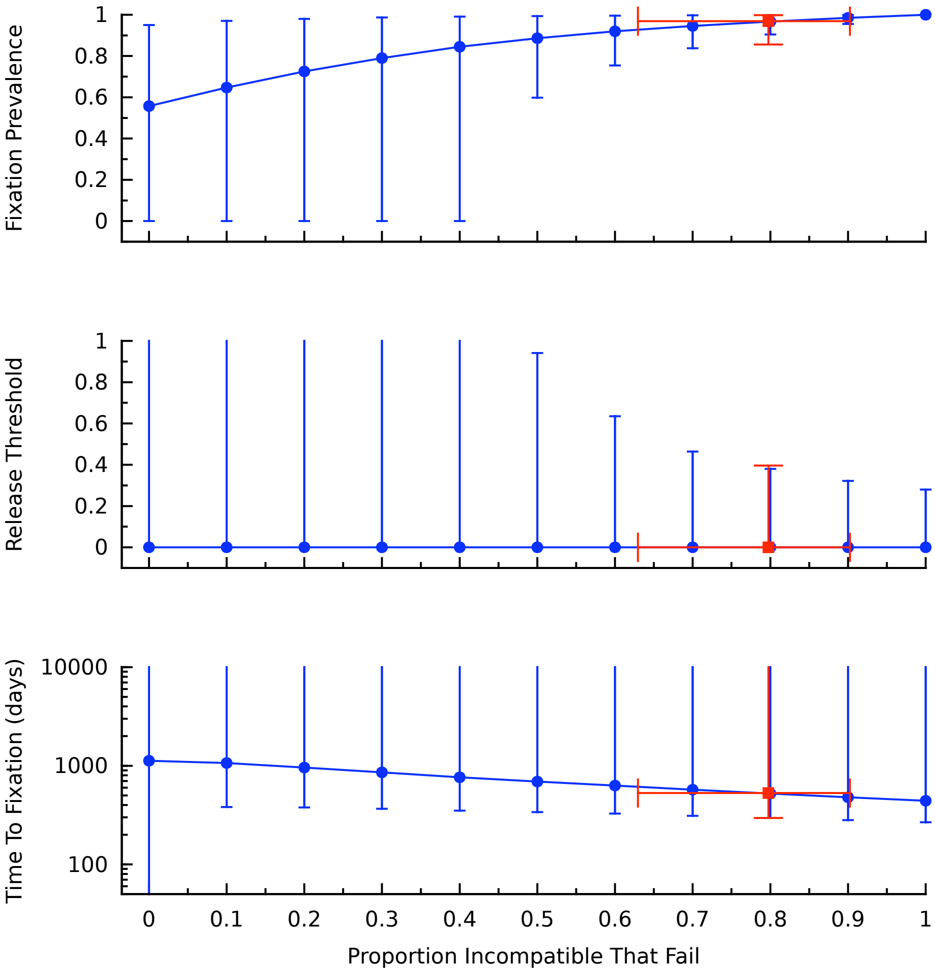

Figure 8: Fixation prevalence (p_F), release threshold (q_T) and time to fixation (τ) vs. proportion of crosses between a *Wolbachia*-positive sperm and *Wolbachia*-negative eggs where the egg fails to fertilize (s_h). The red box and bars show the median and 95% credible interval for the baseline parameter values, with the horizontal bars showing the credible interval of the parameter from the MCMC estimation and the vertical bars showing the credible interval for time to fixation, while the blue circles and bars show the median 95% credible interval as the parameter is varied.

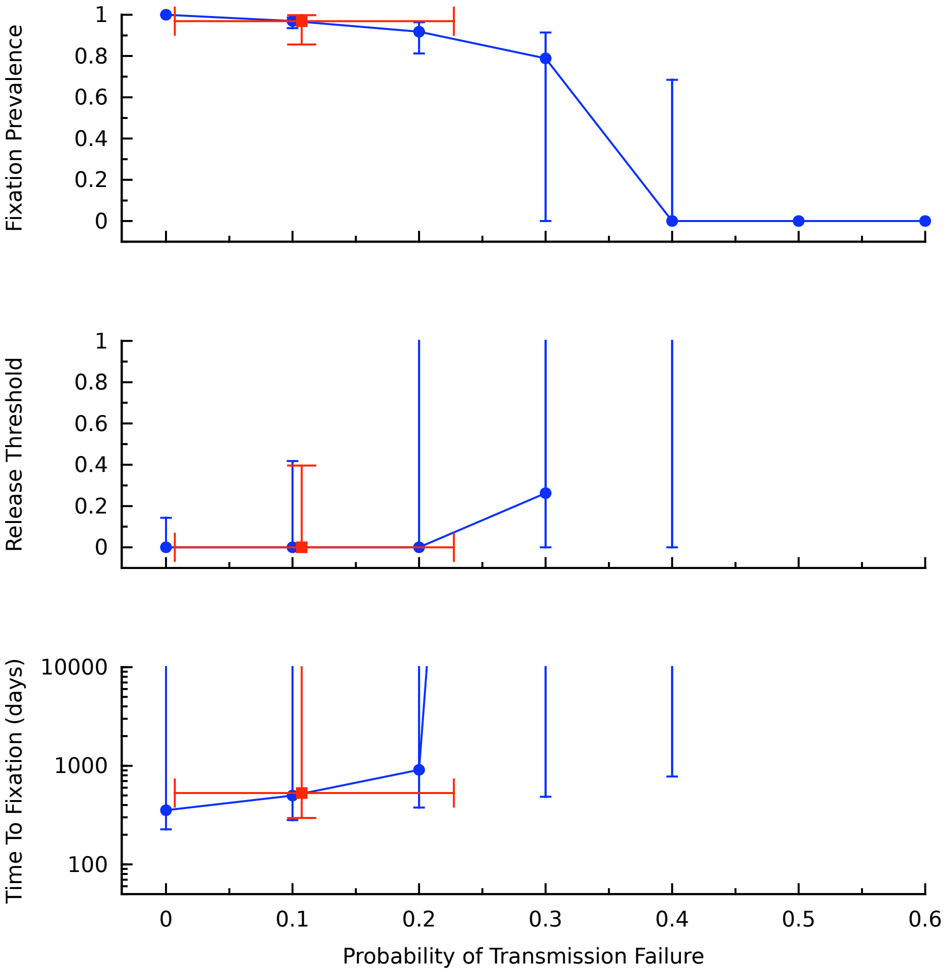

Figure 9: Fixation prevalence (p_F), release threshold (q_T) and time to fixation (τ) vs. probability of failure for *Wolbachia* transmission from mother to offspring (μ). The red box and bars show the median and 95% credible interval for the baseline parameter values, with the horizontal bars showing the credible interval of the parameter from the MCMC estimation and the vertical bars showing the credible interval for fixation prevalence, release threshold, or time to fixation, while the blue circles and bars show the median 95% credible interval as the parameter is varied.

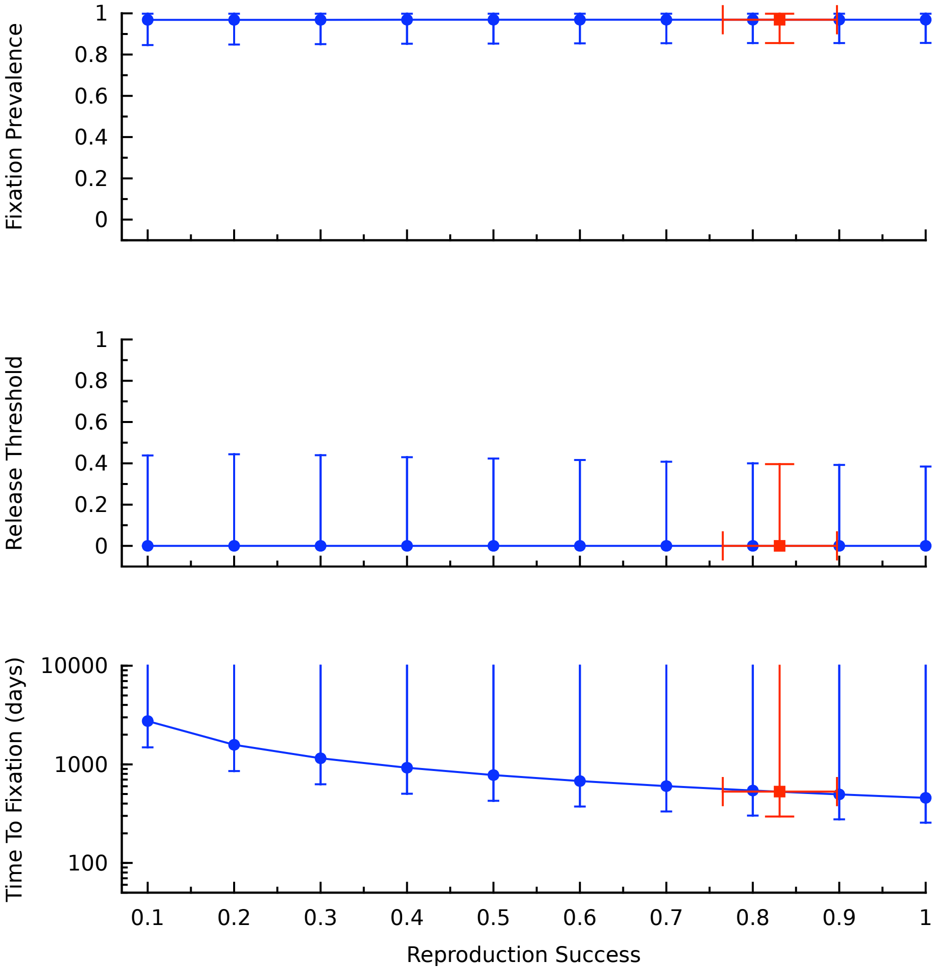

Figure 10: Fixation prevalence (p_F), release threshold (q_T) and time to fixation (τ) vs. reproductive success for wild-type tsetse (f_W). The red box and bars show the median and 95% credible interval for the baseline parameter values, with the horizontal bars showing the credible interval of the parameter from the MCMC estimation and the vertical bars showing the credible interval for release threshold or time to fixation, while the blue circles and bars show the median 95% credible interval as the parameter is varied.

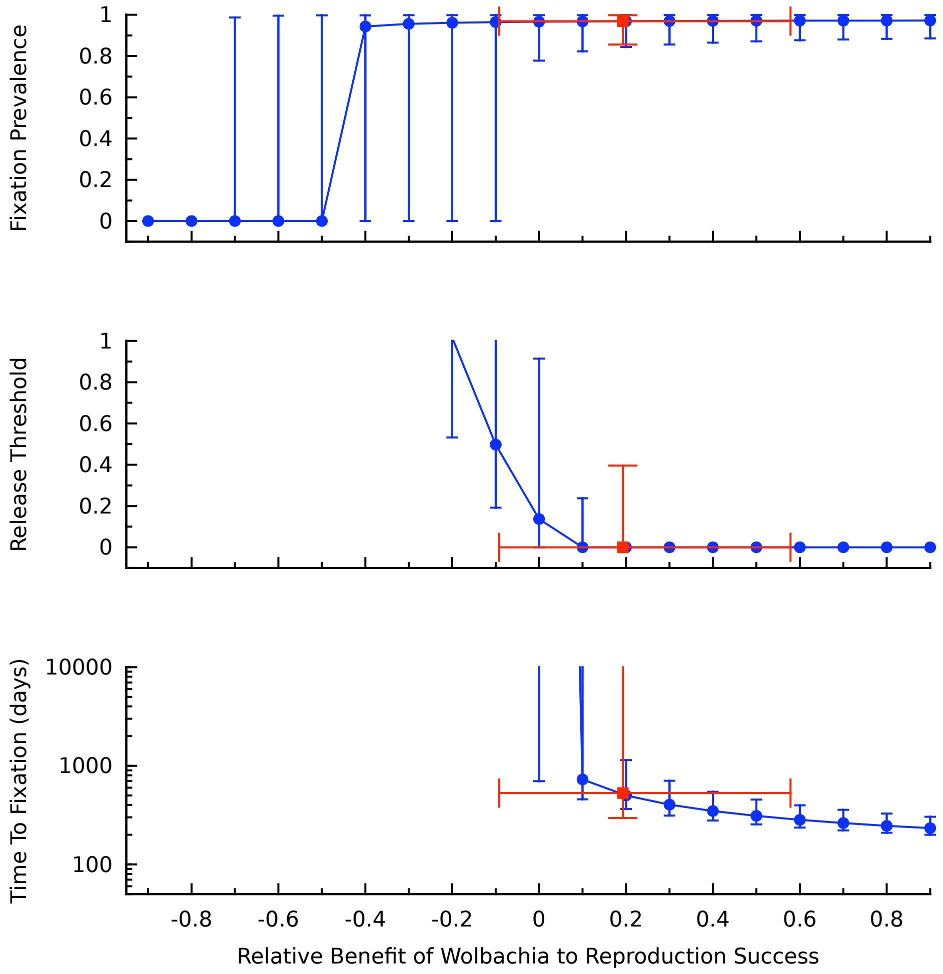

Figure 11: Fixation prevalence (p_F), release threshold (q_T), and time to fixation (τ) vs. relative fecundity benefit of *Wolbachia* infection ($s_{f,\mathrm{Wol}}$). The red box and bars show the median and 95% credible interval for the baseline parameter values, with the horizontal bars showing the credible interval of the parameter from the MCMC estimation and the vertical bars showing the credible interval for fixation prevalence, release threshold, or time to fixation, while the blue circles and bars show the median 95% credible interval as the parameter is varied.

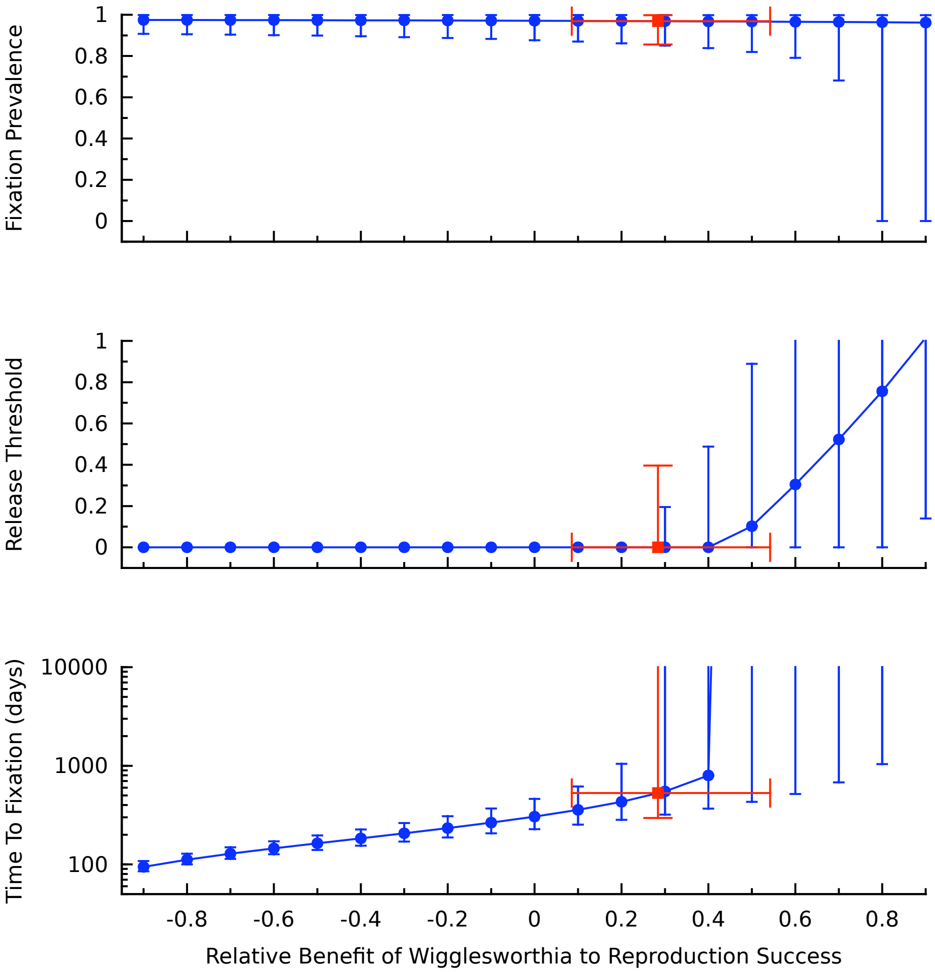

Figure 12: Fixation prevalence (p_F), release threshold (q_T), and time to fixation (τ) vs. relative fecundity benefit of *Wigglesworthia* infection ($s_{f,\mathrm{Wig}}$). The red box and bars show the median and 95% credible interval for the baseline parameter values, with the horizontal bars showing the credible interval of the parameter from the MCMC estimation and the vertical bars showing the credible interval for fixation prevalence, release threshold, or time to fixation, while the blue circles and bars show the median 95% credible interval as the parameter is varied.

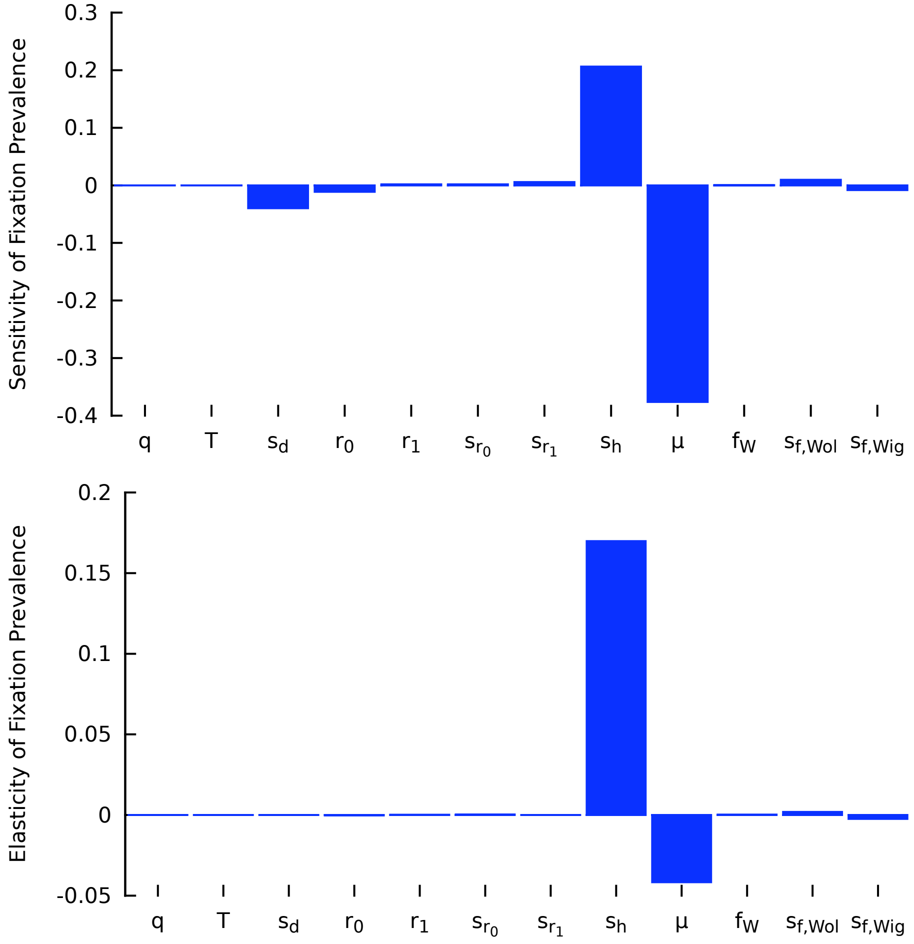

Figure 13: Sensitivity and elasticity of fixation prevalence.

by a relative increment of 1% of their baseline values, while the parameters with zero as the baseline value were varied by the absolute increment of 0.01. Each parameter was both increased and decreased by its increment and the median fixation prevalence and median time to fixation were then calculated over the sample parameter set from the MCMC estimation. Fixation prevalence showed strongest sensitivity and elasticity to proportion of incompatible crosses that fail (s_h) and to transmission failure (μ) and relatively little sensitivity and elasticity to the other parameters (Figure 13). Time to fixation showed substantial sensitivity and elasticity to a broader range of parameters (Figure 14).

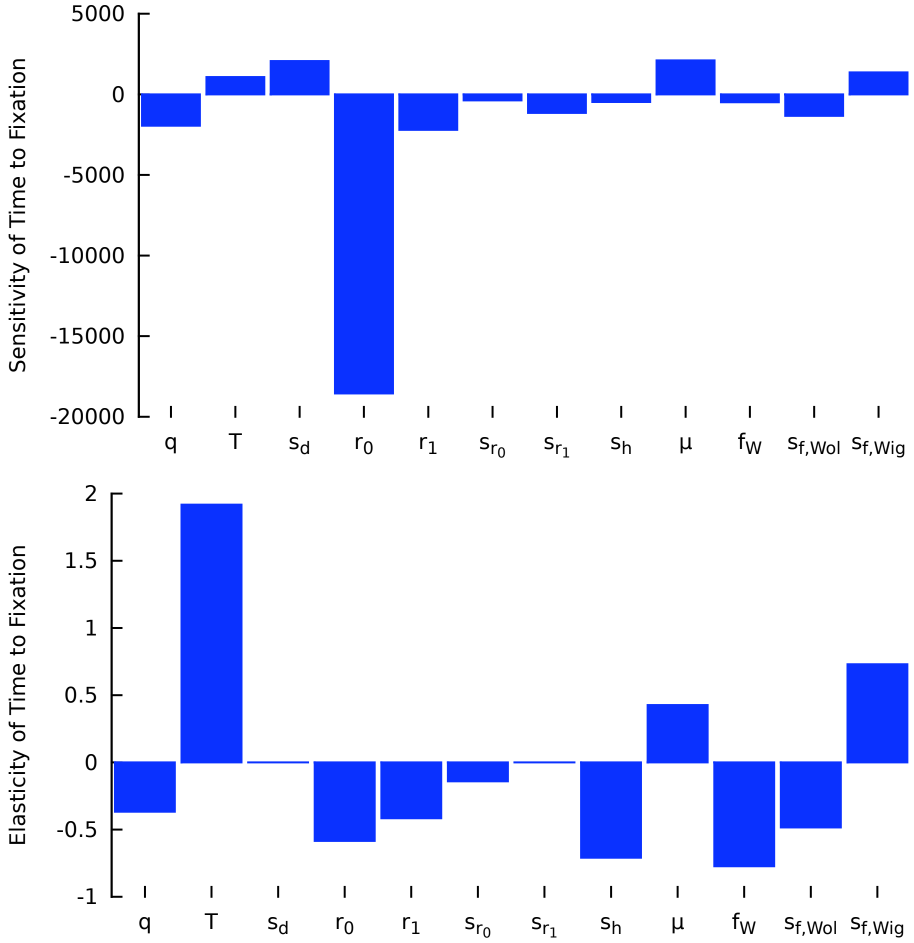

Figure 14: Sensitivity and elasticity of time to fixation.

References

[1] H. Caswell. *Matrix Population Models*. Sinauer, Sunderland, Massachusetts, USA, 2001. ISBN 0-87893-096-5.

[2] C. F. Curtis. Selfish genes in mosquitoes. *Nature*, 357:450, 1992.

[3] A. Gelman, J. B. Carlin, H. S. Stern, and D. B. Rubin. *Bayesian Data Analysis*. Chapman & Hall, New York, 1995. ISBN 0-412-03991-5.

[4] A. Hoffmann, M. Turelli, and L. Harshman. Factors affecting the distribution of cytoplasmic incompatibility in *Drosophila simulans*. *Genetics*, 126(4):933–948, 1990.

[5] S. Leak. *Tsetse Biology and Ecology: Their Role in the Epidmiology and Control of Trypanosomosis*. CABI Publishing, New York, 1999. ISBN 0-85199-300-1.

[6] M. Turelli and A. Hoffmann. Rapid spread of an inherited incompatibility factor in california *Drosophila*. *Nature*, 353(6343):440–442, 1991.

[7] M. Turelli and A. Hoffmann. Microbe-induced cytoplasmic incompatibility as a mechanism for introducing transgenes into arthropod populations. *Insect Molecular Biology*, 8(2):243–255, 1999.
